# A New Species of *Ultratenuipalpus* (Acari: Tenuipalpidae) from Brazil and Re-Description of *Ultratenuipalpus meekeri* (De Leon), the Type Species of the Genus, with DNA Barcodes †

**DOI:** 10.3390/ani13111838

**Published:** 2023-06-01

**Authors:** Elizeu B. Castro, Jennifer J. Beard, Ronald Ochoa, Gary R. Bauchan, Gabriel Otero-Colina, Ashley P. G. Dowling, Antonio C. Lofego, Reinaldo J. F. Feres

**Affiliations:** 1 Graduate Program in Biodiversity, Department of Biological Sciences, São Paulo State University (UNESP), São José do Rio Preto 15054-000, SP, Brazil; 2 Queensland Museum, P.O. Box 3300, South Brisbane, QLD 4101, Australia; 3 Systematic Entomology Laboratory (SEL), Agricultural Research Service (ARS), United States Department of Agriculture (USDA), Beltsville Agricultural Research Centre (BARC), Beltsville, MD 20705, USA; 4 Electron and Confocal Microscopy Unit (ECMU), Agricultural Research Service (ARS), United States Department of Agriculture (USDA), Beltsville Agricultural Research Centre (BARC), Beltsville, MD 20705, USA; 5 Posgrado en Entomología y Acarología, Colegio de Postgraduados, Texcoco 56264, Estado de Mexico, Mexico; 6 Department of Entomology and Plant Pathology, University of Arkansas, Fayetteville, AR 72704, USA; 7 Department of Biological Sciences, Institute of Biosciences, Humanities and Exact Sciences (IBILCE), São Paulo State University (UNESP), São José do Rio Preto 15054-000, SP, Brazil

**Keywords:** flat mites, new species, ferns, integrative taxonomy, LT-SEM, COI, ontogeny

## Abstract

**Simple Summary:**

The flat mite family Tenuipalpidae includes 41 genera and more than 1100 species worldwide, and is considered one of the most important families of phytophagous mites. The *Ultratenuipalpus* is a small genus with 25 known species present in almost all zoogeographic regions. Here, a new species *Ultratenuipalpus parameekeri* Castro, Ochoa & Feres sp. nov. is described from specimens collected on ferns from Brazil. It represents the first species of the genus described from the country. The type species of the genus *Ultratenuipalpus meekeri* (De Leon) is redescribed based on types and newly collected material from Mexico. Highly detailed low-temperature scanning electron image (LT-SEM) micrographs and DNA barcodes are provided for both species. The taxonomy of the genus *Ultratenuipalpus* and the ontogenetic additions of leg setae are discussed.

**Abstract:**

Species of the genus *Ultratenuipalpus* bear a broad subquadrate propodosoma with many large, flattened, lanceolate to ovate dorsal setae. They also bear some plesiomorphic character states, such as the presence of three pairs of ventral *ps* setae. Here, we describe *Ultratenuipalpus parameekeri* Castro, Ochoa & Feres sp. nov. based on adult females, males, and immatures, collected on ferns from Brazil. We also re-describe *Ultratenuipalpus meekeri* (De Leon), the type species of the genus, based on types and newly collected material from Mexico, and include additional novel data (e.g., dorsal and ventral ornamentation, leg chaetotaxy, and setal measurements) in a standardized form. We include highly detailed images obtained using LT-SEM, accompanied by DNA barcodes, for both species. The ontogenetic additions of leg chaetotaxy are presented and discussed.

## 1. Introduction

The *Ultratenuipalpus* Mitrofanov is a small genus of the family Tenuipalpidae (Trombidiformes: Tetranychoidea), with 25 known species to date [1,2,3]. Most species are described from three countries: New Zealand (10 species), Australia (3), and Chile (3) [3,4]. Species of the genus bear a broad subquadrate propodosoma with many large, flattened, lanceolate, and/or obovate to ovate dorsal setae [2]. They also bear some potentially plesiomorphic character states, such as the presence of three pairs of pseudanal setae (*ps1–ps3*) and the absence of genital plates [1,2], which are both shared across the Tetranychoidea [5].

The *Ultratenuipalpus* shares many character states with the genera *Extenuipalpus* and *Tenuipalpus*, such as having the prodorsum broader than the opisthosoma [2,6]. The *Ultratenuipalpus* and *Extenuipalpus* also share the character of dorsal opisthosomal setae *h2* not being flagellate; the *Ultratenuipalpus* and *Tenuipalpus sensu stricto* share the presence of lateral body projections associated with prodorsal setae *sc2* [2,6].

The genus *Extenuipalpu*s was recently reinstated and includes only three species described from South Africa [2,3], while *Tenuipalpus* is the largest genus in the flat mite family, with over 300 described species worldwide [3]. According to Beard et al. [2], the *Ultratenuipalpus* and these two genera are closely allied, and the *Extenuipalpus* may occupy a position intermediate between the *Ultratenuipalpus* and *Tenuipalpus*.

Species of the *Ultratenuipalpus* occur on different families of ferns (e.g., Dennstaedtiaceae, Thelypteridaceae), conifers (e.g., Araucariaceae, Podocarpaceae), monocots (e.g., Arecaceae, Asteliaceae), and dicots plants (e.g., Proteaceae, Rubiaceae) [3,4]. While most species have only ever been recorded from the original host plant, there are some species (e.g., *U. aberrans*, *U. coprosmae*) that have been found on multiple plants of different families.

Here, we describe a new species of the *Ultratenuipalpus* collected on ferns from Brazil and re-describe the type species of the genus, *U. meekeri* (De Leon), in a standardized form. As these two species are morphologically similar and share several character states, including the shape of dorsal setae and chaetotaxy of the legs, molecular analyses were undertaken to confirm their separation using material freshly collected from Brazil and Mexico.

## 2. Materials and Methods

A portion of the samples collected of each species was maintained in 70% ethanol for subsequent use in low temperature SEM (LT-SEM) studies. Mites for LT-SEM were studied using the previously described methodology [7]. Another portion of the samples of each species was maintained in 100% alcohol for a subsequent molecular analysis.

DNA was extracted from individual mites using the QIAGEN DNeasy Blood & Tissue kits following standard protocols with the following exceptions: (1) mite specimens were carefully pierced with a sterilized minutin pin and then incubated overnight in a solution of buffer ATL and Proteinase K as per instructions and (2) a final elution was performed with 100 μL to increase the total DNA concentration. A portion of cytochrome oxidase I was amplified by PCR with previously published primers [8,9]. The amplification reactions were performed in 25 μL volumes containing 2.5 μL of manufacturer supplied buffer, 0.2 μL (5 units) of Platinum Taq polymerase (Invitrogen), 2.5 μL dNTP (0.25 mM of each base), 1 μL of each primer (10 mM), 2.5 μL of MgCl_2_ (25 mM), 11.3 μL of ddH_2_O, and 4 μL of the template DNA. The samples were denatured at 94 °C for 2 min, followed by 30 cycles of 1 min denaturation at 92 °C, 1 min annealing at 50 °C, and 1.5 min extension at 72 °C, with a final elongation of 10 min after the completion of all cycles. PCR products were visualized on a 1% agarose gel saturated with GelRed (Biotium, Hayward, CA, USA). DNA was then purified with a QIAquick PCR Purification kit (Qiagen, Germantown, MD, USA). The amplified fragments were sent to Macrogen USA for sequencing. No additional primers were used for sequencing. COI sequences have been deposited in GenBank (https://www.ncbi.nlm.nih.gov/, accessed on 15 January 2023).

All measurements are given in micrometers (μm). Measurements are presented for the holotype followed by the range for all types in parentheses. The number of leg setae is written as the total number of setae followed by the number of solenidia in parentheses. Terminology of leg and body setation is adapted from [10,11,12]. Photographs of slide-mounted specimens were obtained using a Zeiss Axioscope™ microscope (Carl Zeiss Inc., Thornwood, NY, USA) with a differential interference contrast (DIC) 100× Plan Apochromatic objective with an NA 1.4.

Type specimens and vouchers of non-type specimens are deposited in the Collection of Acari, Departamento de Zoologia e Botânica, UNESP, São José do Rio Preto, State of São Paulo, Brazil (DZSJRP, http://www.splink.cria.org.br, accessed on 10 December 2022) and in the National Insect and Mite Collection, National Museum of Natural History, Smithsonian Institution, located at the Systematic Entomology Laboratory (SEL), USDA, Beltsville, MD, USA (NMNH). The holotype of *U. meekeri* is deposited in the Museum of Comparative Zoology (MCZ), Harvard University, Cambridge, MA, USA.

## 3. Results

### 3.1. Description of Ultratenuipalpus parameekeri Castro, Ochoa & Feres sp. nov.

Family Tenuipalpidae Berlese, 1913
Genus *Ultratenuipalpus* Mitrofanov, 1973
Type species: *Ultratenuipalpus meekeri* (De Leon), 1957

Diagnosis of the genus (Based on [2]). “Body shape from elongate-ovate to broadly rounded; broad propodosoma differentiated from narrower opisthosoma (although anterior opisthosoma is broad at junction with propodosoma). Anterior margin of prodorsum usually with median forked projection forming a short notch.” Prodorsum with one pair of lateral body projections anterior to setae *sc2* present or absent. Posterior margin of opisthosoma with a broad rounded projection between setae *h1* usually present. “Prodorsal shield divided by two oblique folds running from vicinity of the eyes angled medially to posterior margin of shield, superficially dividing the shield into three smaller plate-like regions; a small plate is indicated between setae *c3*–*d3* on dorsal opisthosomal margin; posteroventral body margin often with band of pustulate cuticle. Dorsal opisthosoma with setae *c1*, *c3*, *d1*, *d3*, *e1*, *e3*, *f3*, *h1*, *h2* present (except setae *f3* absent in *U. aberrans*); setae *f2* present or absent, when present then inserted on lateral margin aligned with lateral setae *e3*, *f3*, *h1*, *h2*; setae *c2*, *d2*, *e2* absent. Setae *h2* not flagellate, similar in form to *h1*; setae *sc2*, *e3*, *f2*, *f3*, *h1*, *h2* flattened, lanceolate, oblanceolate, obovate to ovate, with *sc2* often falcate; form of other dorsal setae variable (e.g., *sc2* and *f3* flagellate in *U. bunyai*). Three pairs of *ps* (pseudanal) setae present; female with ps3 positioned anteriorly on anal valves and much shorter than *ps1*–*2*, which are closely associated with each other and positioned posterolaterad anal valves; setae *ps2* usually much longer than *ps1*; male with ps3 modified into accessory genital stylets and inserted on elongate genitoanal valves, with *ps1*–*2* positioned as in female. Ventral, genital and anal regions membranous, without defined sclerotized plates; flap of ovipore and anus surrounded by strongly plicate and wrinkled membranous cuticle. Genital setae *g2* inserted slightly anterior to *g1* on reduced genital flap; *g1*–*2* often aligned longitudinally with setae *ag*. Intercoxal setae (*3a*, *4a*) not multiplied. Palps four segmented; palp tibiae with 1–2 setae; palp tarsi with 1–3 phaneres, with solenidion always present, sometimes curved, often inserted basally on palp tarsus segment at junction with palp tibia. Dorsal setae on legs inserted in lateral position. Femora of legs I–II with four setae (*d*, *l*’, *v’*, *bv″*); genua I–II with three setae (*d*, *l’*, *l″*) (except some species variously described with two setae—*U. acharis* (genua I–II with 3–2 setae; possibly *d* absent), *U. pterophilus* (genua I–II with 2–2)); tibiae I–II usually with five setae (except two species described with four setae, *U. avarua* (*v″* absent) and *U. lacorpuzrarosae* (possibly *d* absent)). Tarsal claws pad-like. Immature stages with setae *c1* inserted distinctly anterior to level of setae *c3*. See also diagnosis of [1].”

Description

Diagnosis. Female: As per genus, in addition to: prodorsal setae *v2*, *sc1* minute to short, and *sc2* large, flattened obovate to ovate; dorsal opisthosoma with 10 pairs of setae (*f2* present); most of the dorsal opisthosomal setae large, flattened, obovate to ovate, except setae *d3* is distinctly short and *c3* almost orbicular; pair lateral body projections anterior to setae *sc2* and projection between opisthosomal setae *h1* both present; palp four segmented, setal formula 0, 0, 2, 2. Male: Opisthosoma narrower than that of the female, with distinct transverse constriction (waist) between setae *d1* and *e1*; many dorsal setae similar in form to those of females, except *c1*, *d1*, and *e1* short to minute, *d3* longer, and setae along posterior margin of opisthosoma (especially *e3*) narrower and more elongated than those of the female. Tarsi I–II each with two solenidia (ω′ paraxial and ventrolateral; ω” antiaxial); tarsus III with one solenidion ω′ paraxial and ventrolateral. Immatures: with lateral body projection anterior to setae *sc2* present (except absent in larvae); posterior projection between setae *h1* absent; dorsal setae similar in general form to those of the female, except setae *c1*, *d1*, and *e1* short to minute. Larvae with central prodorsum and pygidial region of posterior opisthosoma with finely colliculated integument.

Female (*n* = 10) (Figure 1, Figure 2, Figure 3, Figure 4, Figure 5, Figure 6, Figure 7, Figure 8, Figure 9 and Figure 10)

Body measurements: distance between setae *v2*–*h1* 350 (340–375), *sc2*–*sc2* 220 (215–230); other measurements: *v2*–*v2* 45 (37–45), *sc1*–*sc1* 93 (90–110), *c1*–*c1* 60 (55–63), *c3*–*c3* 260 (245–265), *d1*–*d1* 40 (37–45), *d3*–*d3* 240 (230–240), *e1*–*e1* 32 (27–33), *e3*–*e3* 220 (215–225), *f2*–*f2* 205 (205–215), *f3*–*f3* 170 (170–185), *h1*–*h1* 58 (58–68), *h2*–*h2* 120 (115–135).

**Figure 1:**
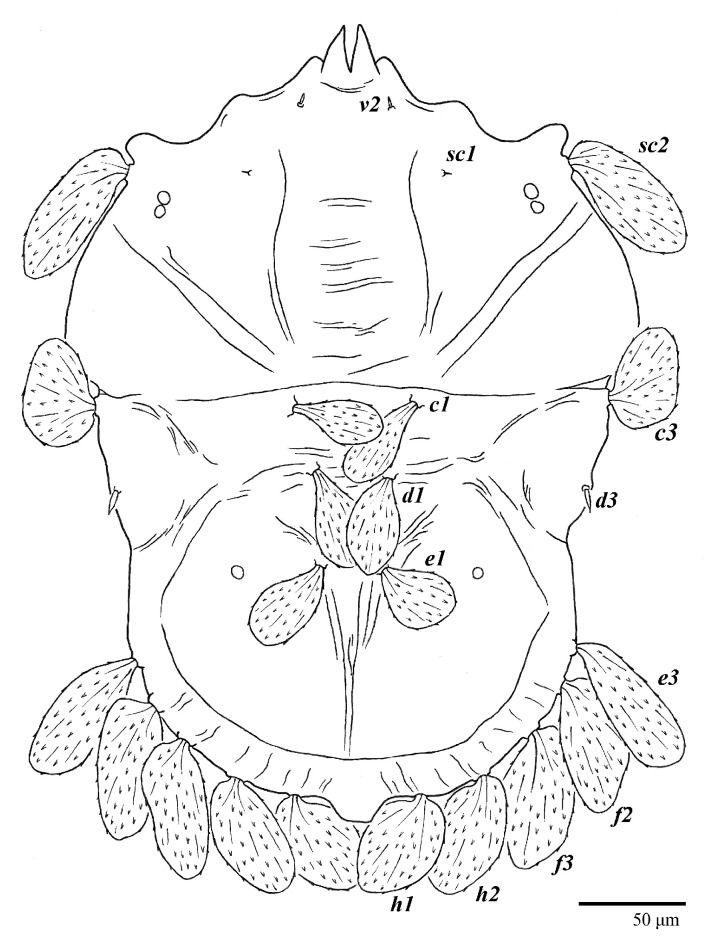
*Ultratenuipalpus parameekeri* Castro, Ochoa & Feres sp. nov. (Female): view of dorsum.

**Figure 2:**
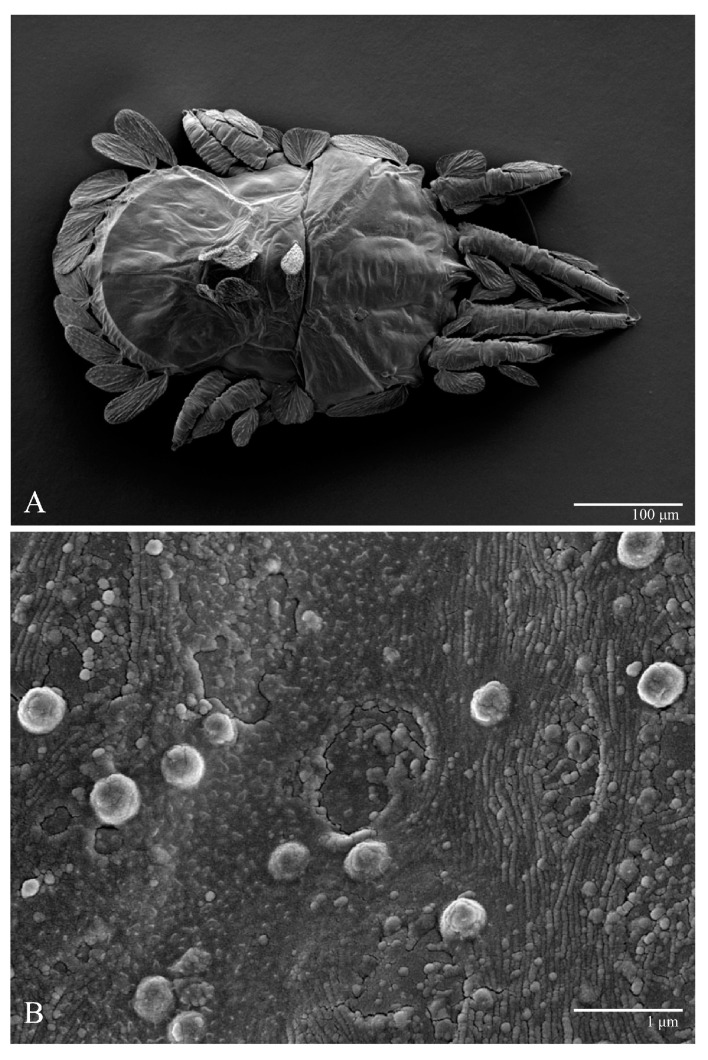
*Ultratenuipalpus parameekeri* Castro, Ochoa & Feres sp. nov. (Female): (**A**) dorsal view; (**B**) view of cuticular microplates on the dorsum.

**Figure 3:**
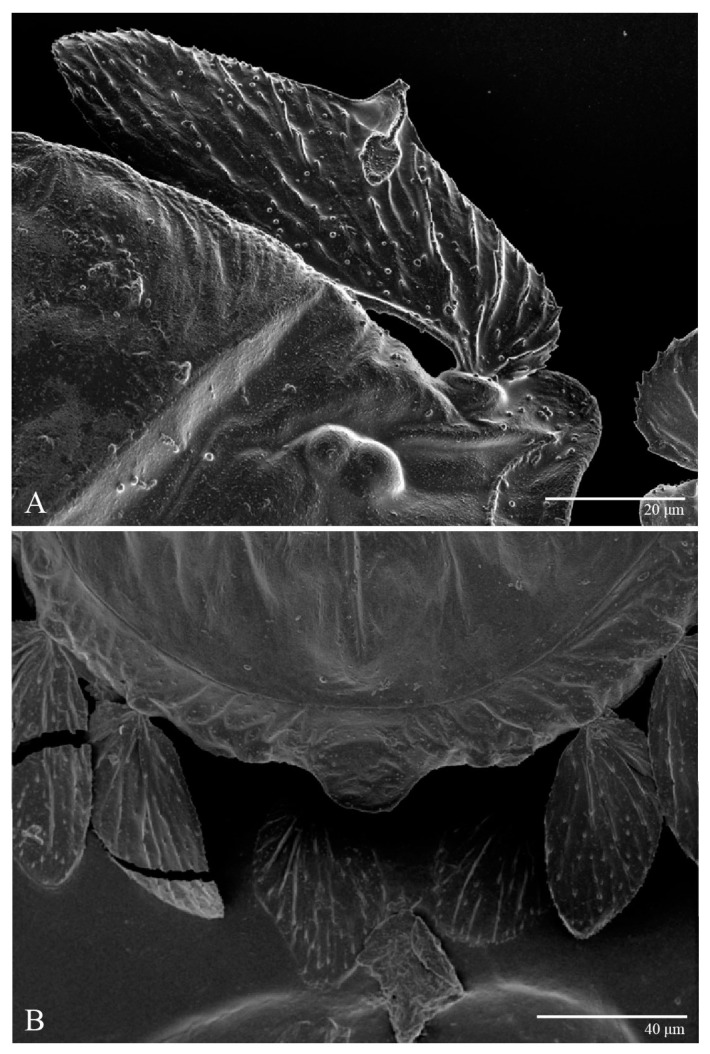
*Ultratenuipalpus parameekeri* Castro, Ochoa & Feres sp. nov. (Female): (**A**) detail of the lateral region of prodorsum, with setae *sc2*. Note the presence of body projection anterior to *sc2*; (**B**) posterior region of opisthosoma, indicating the body projection on posterior margin.

**Figure 4:**
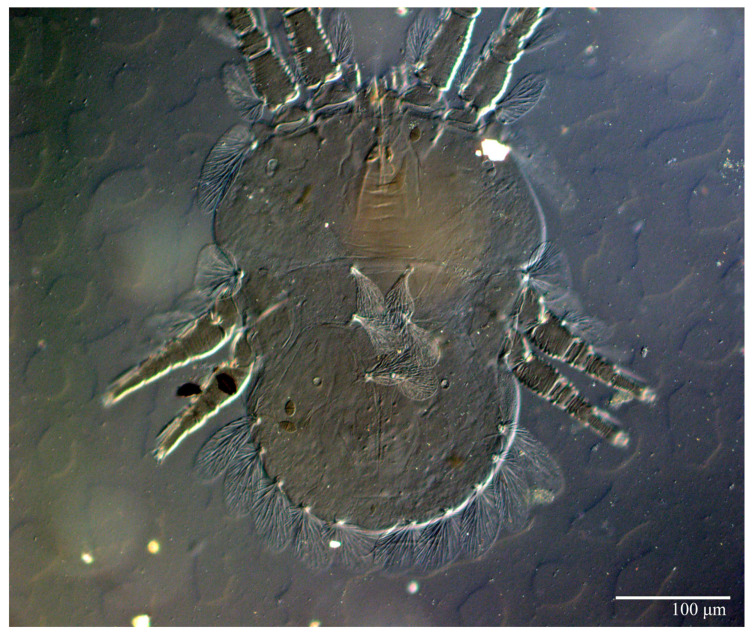
*Ultratenuipalpus parameekeri* Castro, Ochoa & Feres sp. nov. (Female): view of dorsum.

Dorsum (Figure 1, Figure 2, Figure 3 and Figure 4). Anterior margin of prodorsum with a short median forked projection forming a short notch 27 (20–27). Dorsum smooth, with pair lateral projections anterior to setae *sc2* and single projection between opisthosomal setae *h1* (Figure 3B). A pair of converging folds from the eyes to near the sejugal furrow on the prodorsum posterior margin. Prodorsal setae *v2* and *sc1* short to minute; *sc2* large, flattened elongate obovate (Figure 1 and Figure 3A); most opisthosomal setae similar to prodorsal setae *sc2*, except *d3* is short. Setal measurements: *v2* 5 (4–7), *sc1* 3 (3–5), *sc2* 74 (74–82), *c1* 52 (52–58), *c3* 36 (36–45), *d1* 55 (54–55), *d3* 10 (8–10), *e1* 50 (48–55), *e3* 70 (70–81), *f2* 65 (65–70), *f3* 61 (60–68), *h1* 52 (52–60), *h2* 58 (58–65).

**Figure 5:**
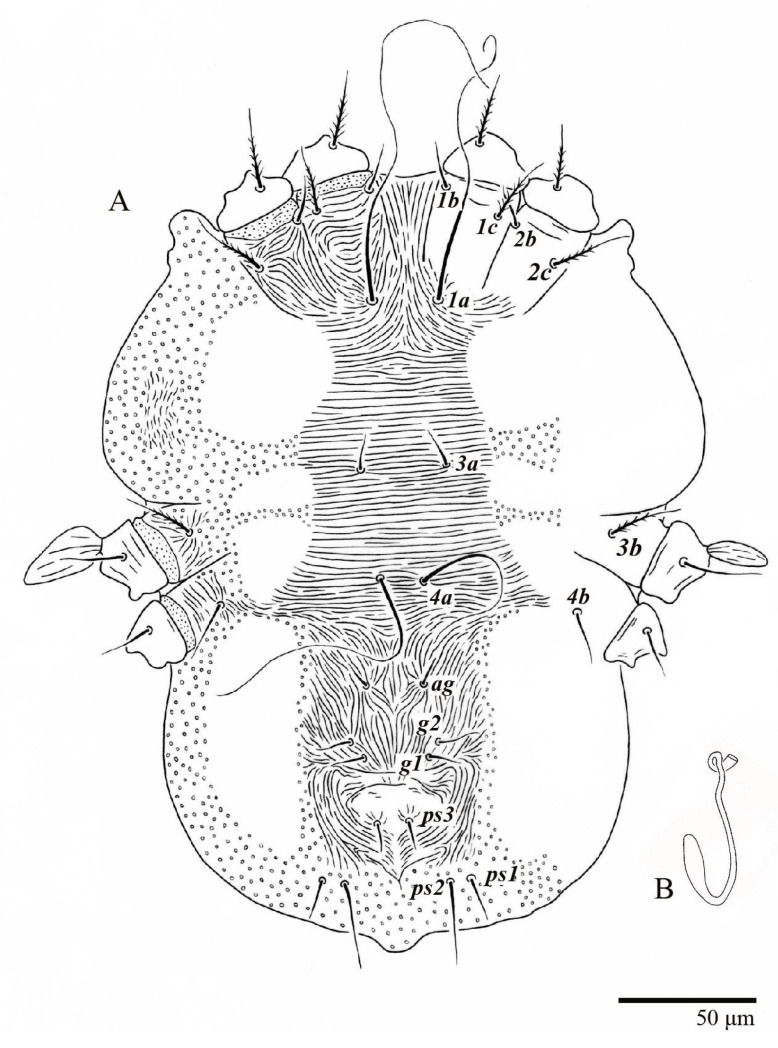
*Ultratenuipalpus parameekeri* Castro, Ochoa & Feres sp. nov. (Female): (**A**) view of venter; (**B**) spermatheca.

**Figure 6:**
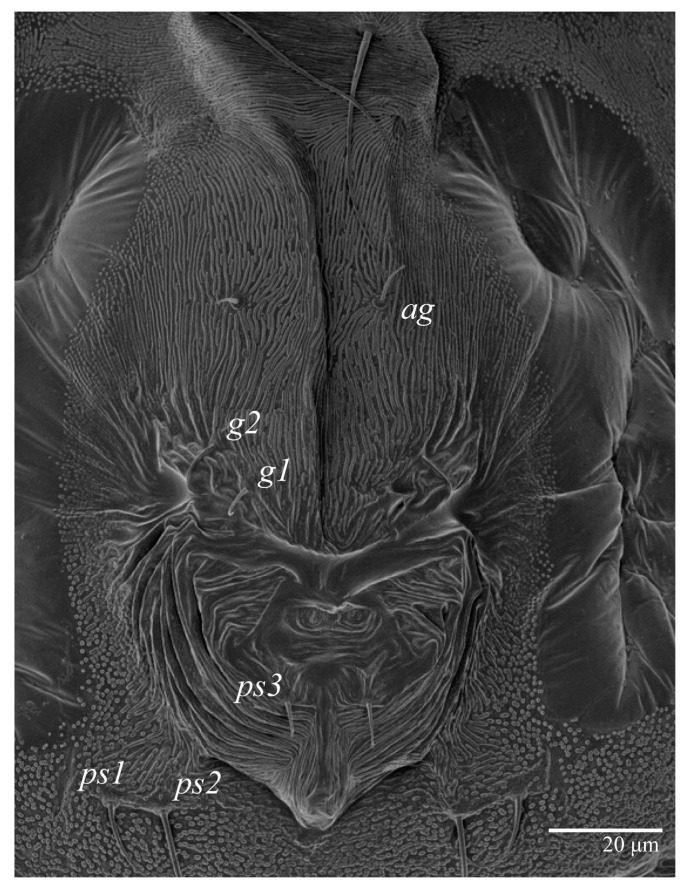
*Ultratenuipalpus parameekeri* Castro, Ochoa & Feres sp. nov. (Female): posterior ventral opisthosoma.

Venter (Figure 5A and Figure 6). Ventral integument weakly striate along central region and densely colliculated around lateral body margin; ventral, genital, and anal plates not developed, and entire region membranous and distinctly plicate; ventral setae filiform, with coxal setae *1c*, *2c*, and *3b* barbed; setae *ps2* distinctly longer than *ps1*. Setal measurements: *1a* 105 (100–135), *1b* 13 (13–16), *1c* 30 (25–30), *2b* 22 (22–26), *2c* 38 (30–39), *3a* 18 (15–18), *3b* 32 (31–36), *4a* 145 (115–145), *4b* 22 (18–22), *ag* 10 (10–11), *g1* 16 (14–16), *g2* 16 (15–18), *ps1* 28 (22–28), *ps2* 53 (50–55), *ps3* 12 (10–13).

**Figure 7:**
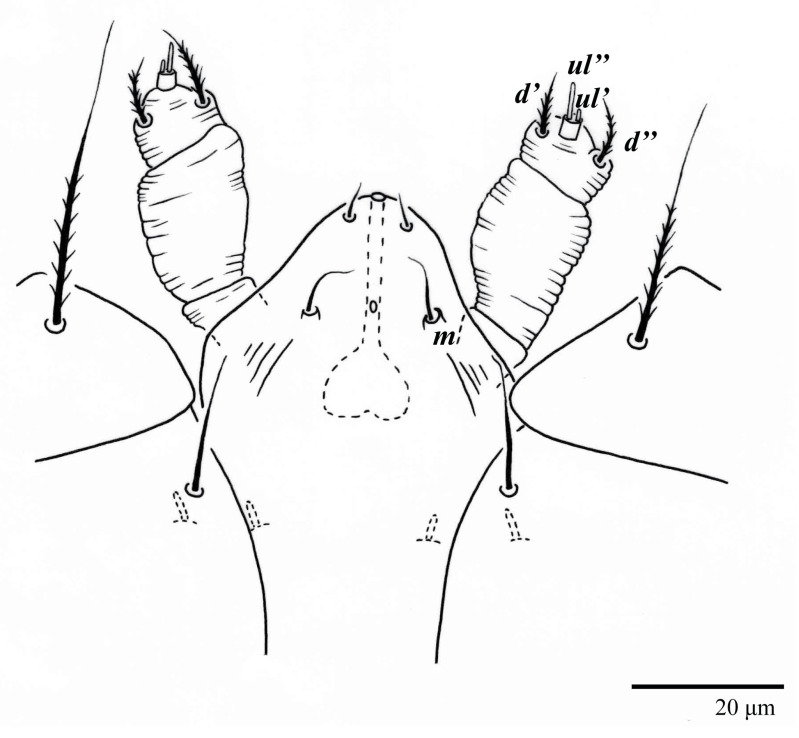
*Ultratenuipalpus parameekeri* Castro, Ochoa & Feres sp. nov. (Female): ventral infracapitulum.

**Figure 8:**
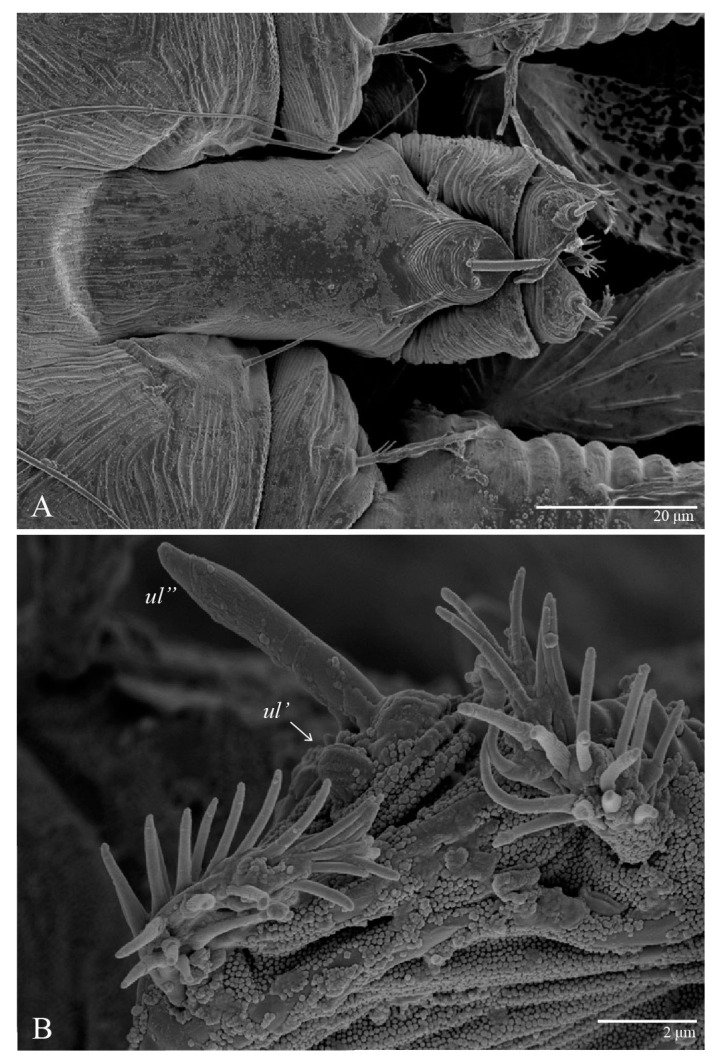
*Ultratenuipalpus parameekeri* Castro, Ochoa & Feres sp. nov. (Female): (**A**) view of ventral infracapitulum; (**B**) detail of palp.

**Figure 9:**
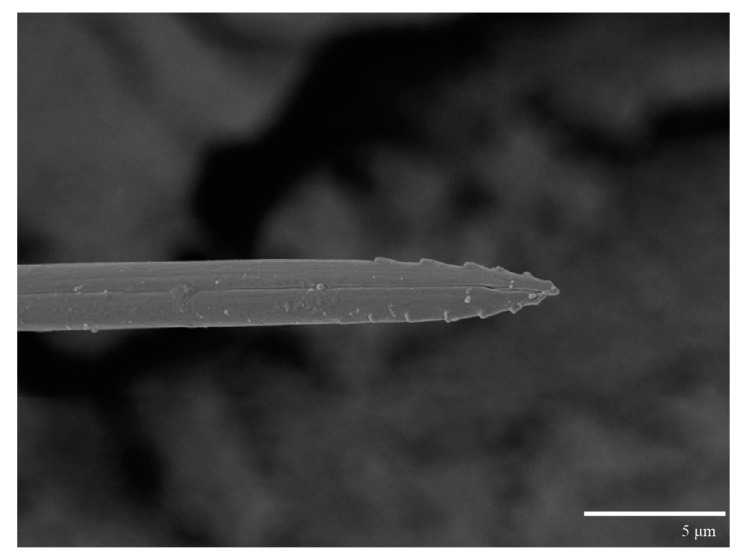
*Ultratenuipalpus parameekeri* Castro, Ochoa & Feres sp. nov. (Female): detail of stylet tip with lateral serrations.

Gnathosoma (Figure 7, Figure 8 and Figure 9). Palps four segmented, setal formula: 0, 0, 2, 2; tibia with two setae, *d′* 7 (6–8), *d″* 6 (5–6), tarsus with one eupathidium 5 (3–5) and one solenidion 1 (1–2). Ventral setae *m* 8 (6–8); distance between setae *m–m* 14 (13–16). Tips of cheliceral stylets with a few bluntly rounded lateral projections (Figure 9).

Spermatheca (Figure 5B). Duct length ca. 75–85, terminating in smooth rounded bulb.

**Figure 10:**
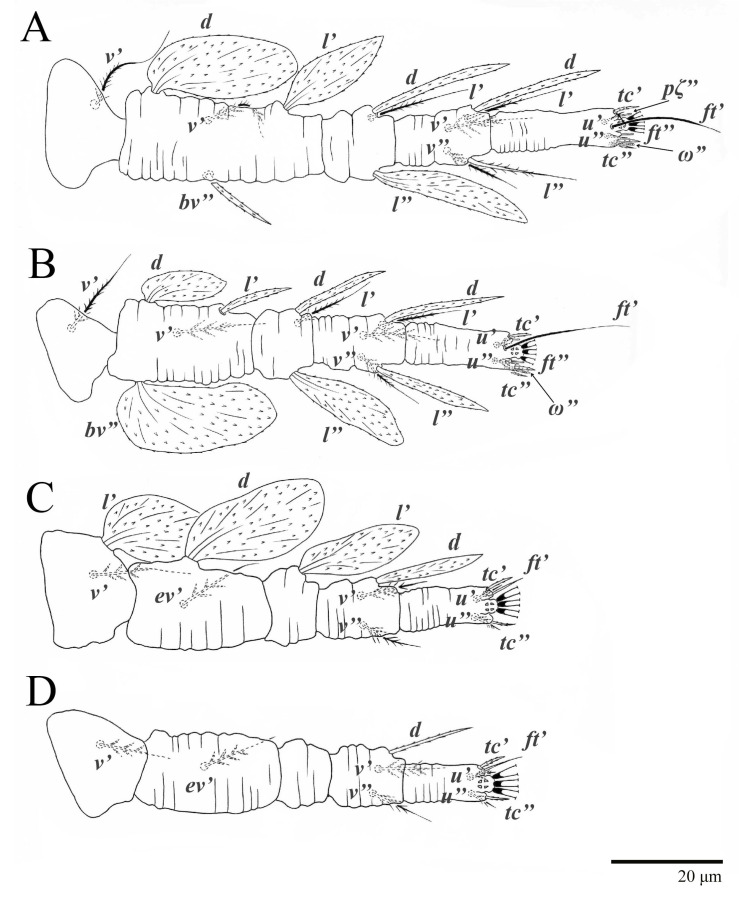
*Ultratenuipalpus parameekeri* Castro, Ochoa & Feres sp. nov. (Female): (**A**) leg I; (**B**) leg II; (**C**) leg III; (**D**) leg IV. (Right legs).

Legs (Figure 10). Setation (from coxae to tarsi): I 3–1–4–3–5–8(1), II 2–1–4–3–5–8(1), III 1–2–2–1–3–5, IV 1–1–1–0–3–5. Tarsi I–II each with one solenidion *ω”* 7 (6–8) (for both tarsi I and tarsi II) and two eupathidia *pζ′–pζ”* (5–6, 5–6; 5, 5–6, respectively); femur I with setae *d* obovate and *l′* broadly lanceolate; femur II with setae *d* narrowly obovate, *l′* lanceolate, and *bv”* obovate to broadly falcate. Femora, genua, and tibiae with setae *d* inserted in lateral position. Detail of the development of leg chaetotaxy in Table 1.

Microplates (Figure 2B). The microplate layer forms a reticulate network of thick ridges covered in small, single, irregularly-shaped wax-like crystals or masses.

Color. The body is mostly orange with the margin of prodorsum and opisthosoma with dark spots, eyes red, and legs orange. The dorsal body setae and leg setae white to translucent.

**Figure 11:**
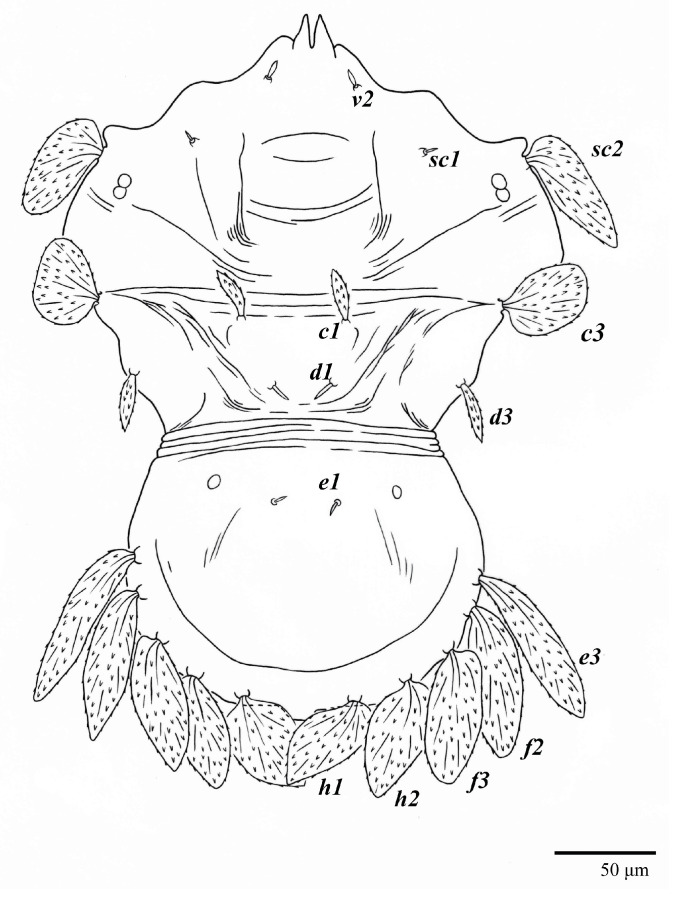
*Ultratenuipalpus parameekeri* Castro, Ochoa & Feres sp. nov. (Male): view of dorsum.

**Figure 12:**
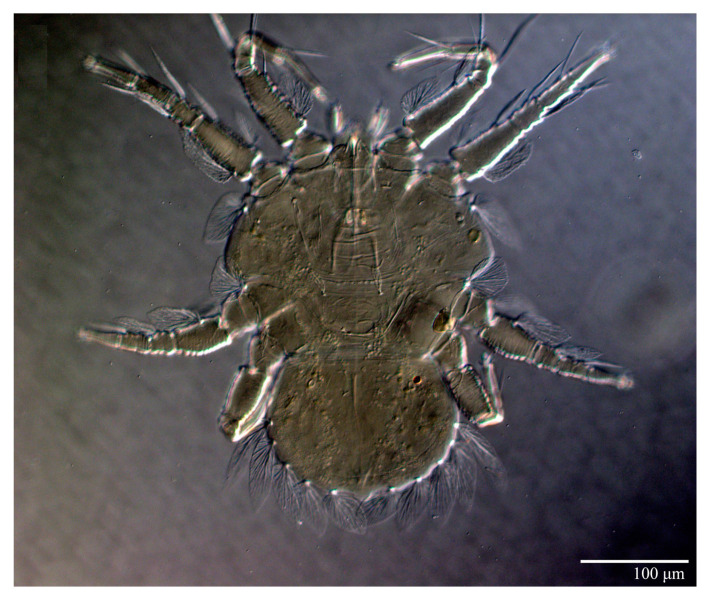
*Ultratenuipalpus parameekeri* Castro, Ochoa & Feres sp. nov. (Male): view of dorsum.

**Figure 13:**
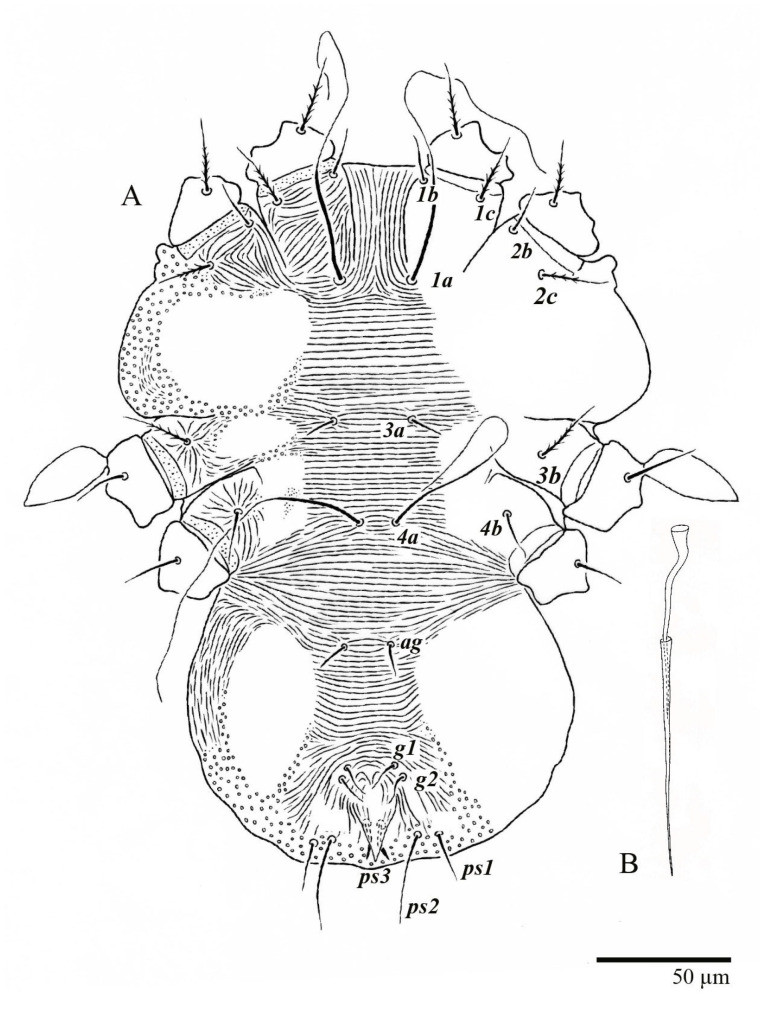
*Ultratenuipalpus parameekeri* Castro, Ochoa & Feres sp. nov. (Male): (**A**) view of venter; (**B**) aedeagus.

**Figure 14:**
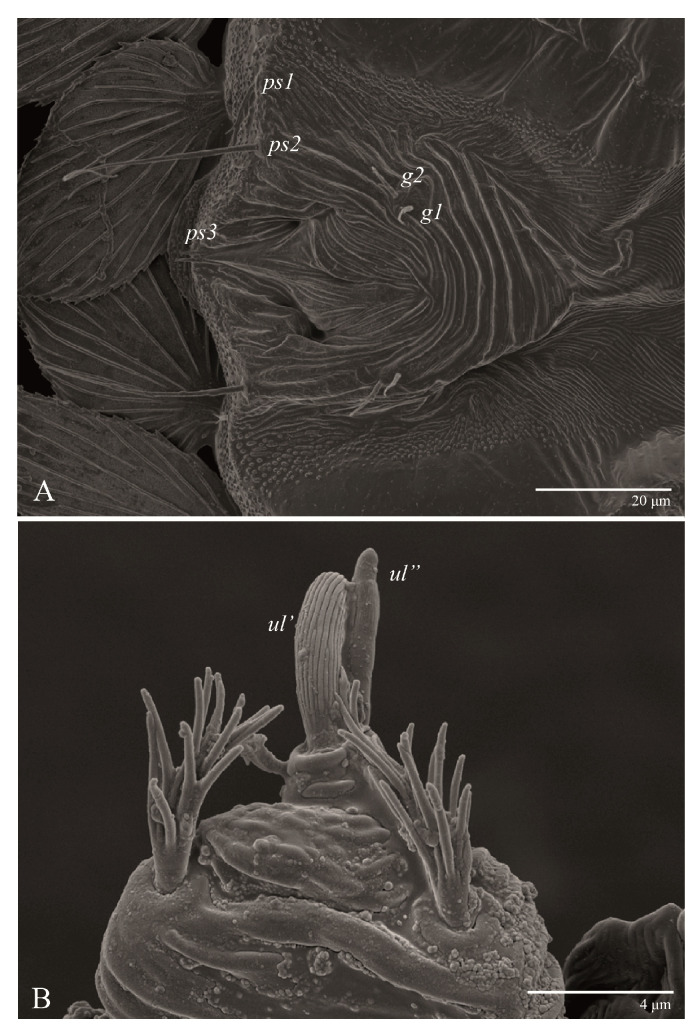
*Ultratenuipalpus parameekeri* Castro, Ochoa & Feres sp. nov. (Male): (**A**) posterior ventral opisthosoma; (**B**) detail of palp. Note the well-developed solenidion.

**Figure 15:**
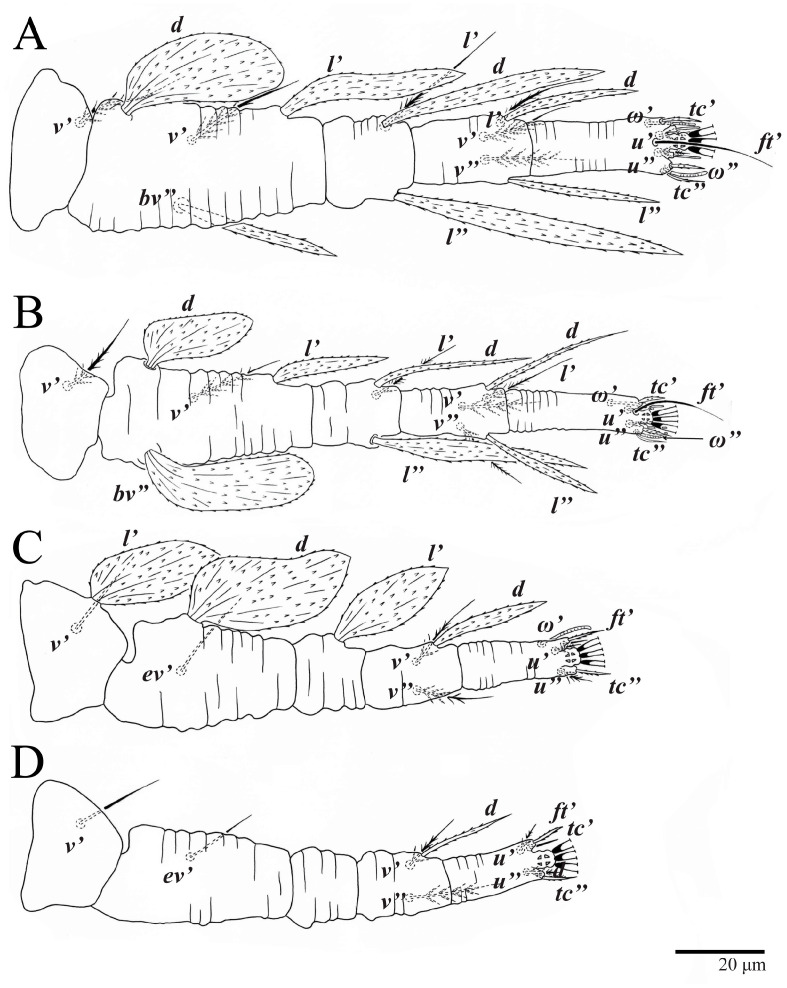
*Ultratenuipalpus parameekeri* Castro, Ochoa & Feres sp. nov. (Male): (**A**) leg I; (**B**) leg II; (**C**) leg III; (**D**) leg IV. (Right legs).

Male (*n* = 5) (Figure 11, Figure 12, Figure 13, Figure 14 and Figure 15)

Body measurements: distance between setae, *v2*–*h1* 285–310, *sc2*–*sc2* 200–220; other measurements: *v2*–*v2* 45–50, *sc1*–*sc1* 105–120, *c1*–*c1* 50–58, *c3*–*c3* 190–215, *d1*–*d1* 27–30, *d3*–*d3* 155–175, *e1*–*e1* 27–33, *e3*–*e3* 165–175, *f2*–*f2* 160–170, *f3*–*f3* 140–145, *h1*–*h1* 55–58, *h2*–*h2* 105–115.

Dorsum (Figure 11 and Figure 12). Anterior margin of prodorsum with a short median forked projection forming a short notch. The dorsum is smooth, with a pair of lateral projections anterior to setae *sc2* and a single projection between opisthosomal setae *h1*. Many dorsal setae similar in general form to those of female, except *c1*, *d1*, and *e1* short to minute, *d3* longer, and setae along posterior margin of opisthosoma (especially *e3*) narrower and more elongated than those of the female. Setal measurements: *v2* 5–7, *sc1* 4–6, *sc2* 60–67, *c1* 29–30, *c3* 40–45, *d1* 8–10, *d3* 19–27, *e1* 5–7, *e3* 77–80, *f2* 60–72, *f3* 60–63, *h1* 49–50, *h2* 53–55.

Venter (Figure 13 and Figure 14A). Ventral integument weakly striate along central region and densely colliculated along lateral body margin; ventral setae filiform, with coxal setae *1c*, *2c*, and *3b* barbed; setae *ps2* distinctly longer than *ps1*; setae *ps3* thickened and inserted ventrally on the elongate tapered anal valves. Setal measurements: *1a* 100–105, *1b* 18–21, *1c* 27–30, *2b* 23–29, *2c* 34–35, *3a* 17–23, *3b* 40–42, *4a* 120–130, *4b* 23–30, *ag* 14–15, *g1* 12–13, *g2* 14–17, *ps1* 21–23, *ps2* 42–55, *ps3* 13–14.

Gnathosoma (Figure 14B). Palps four segmented, setal formula: 0, 0, 2, 2; tibia with two setae, *d′* 7–8, *d″* 7–8, tarsus with one eupathidium 5–6 and one solenidion 6. Ventral setae *m* 6–7; distance between setae *m–m* 13–14.

Legs (Figure 15). Setation (from coxae to tarsi): I 3–1–4–3–5–8(2), II 2–1–4–3–5–8(2), III 1–2–2–1–3–5(1), IV 1–1–1–0–3–5. Tarsi I–II each with two solenidia (one abaxial, one adaxial), tarsi I *ω″* 10–11, *ω′* 16–17, tarsi II *ω″* 11–12*, ω′* 14–15 and two eupathidia *pζ′*–*pζ”* (6–7, 7; 5–6, 5–6), and tarsus III with one solenidion (paraxial and ventrolateral) *ω′* 13–15. Leg setae similar to that of the female; seta *l”* on genu I distinctly elongated. Detail of the development of leg chaetotaxy in Table 1.

Aedeagus (Figure 13B). As figured; ca. 130 long.

**Figure 16:**
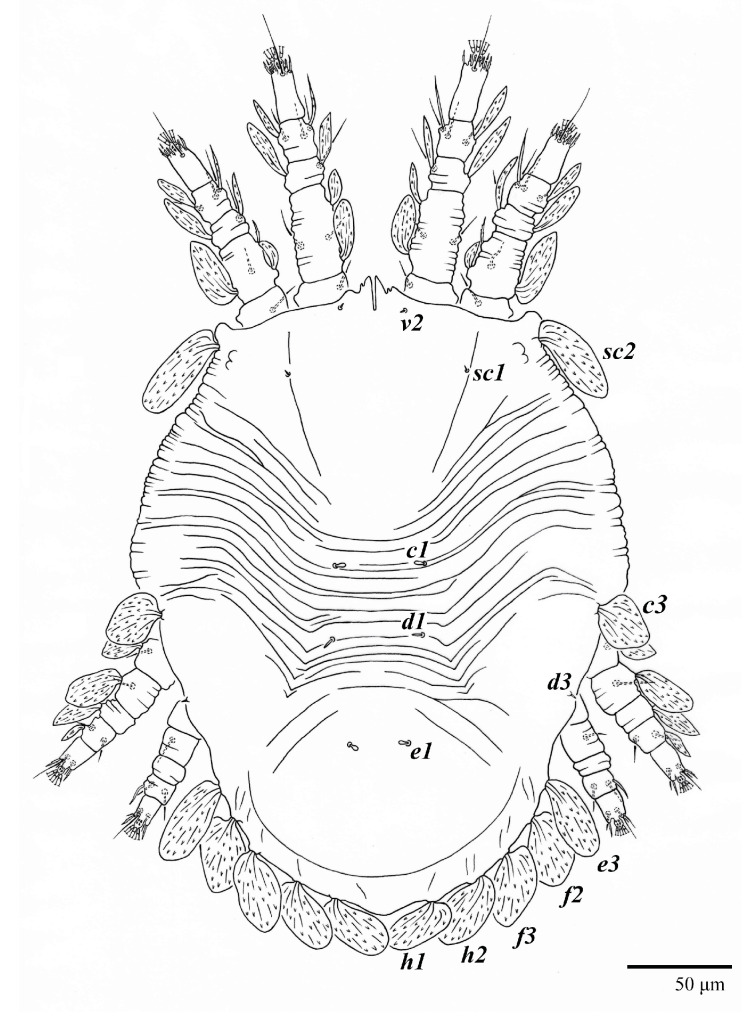
*Ultratenuipalpus parameekeri* Castro, Ochoa & Feres sp. nov. (Deutonymph): dorsum, with detail of legs (unguinal setae *u′–u”* on tarsus I and II are not included in the drawing).

Deutonymph (*n* = 3) (Figure 16)

Body measurements: distance between setae *v2*–*h1* 335–365, *sc2*–*sc2* 165–180; other measurements: *v2*–*v2* 37–40, *sc1*–*sc1* 93–105, *c1*–*c1* 42–55, *c3*–*c3* 235–265, *d1*–*d1* 40–50, *d3*–*d3* 200–215, *e1*–*e1* 18–28, *e3*–*e3* 155–175, *f2*–*f2* 145–160, *f3*–*f3* 120–135, *h1*–*h1* 45–50, *h2*–*h2* 87–95.

Dorsum (Figure 16). Anterior margin of prodorsum with a short median forked projection forming a short notch; pair of lateral projections anterior and adjacent to setae *sc2* present; projection not formed (or rudimentary) between setae *h1*. Prodorsal region smooth; region between setae *sc2*–*c3* with transverse plicae and folds; region posterior to setae *d1*–*d3* smooth. Dorsal setae similar in general form to that of females, except setae *c1*, *d1* and *e1* short to minute. Setal measurements: *v2* 3–4, *sc1* 2–3, *sc2* 60–64, *c1* 3–5, *c3* 32–35, *d1* 2–3, *d3* 3–4, *e1* 3–4, *e3* 45–54, *f2* 40–42, *f3* 41–42, *h1* 35–36, *h2* 38–45.

Gnathosoma. Palps similar to those of female, setal formula: 0, 0, 2, 2; tibia with two setae, *d′* 4–5, *d″* 4–5, tarsus with one eupathidium 3–4 and one minute solenidion, 1 long. Ventral setae *m* 4–5; distance between setae *m–m* 10–11.

Venter. Cuticle covered with fine and mostly transverse striae. Coxal, genital, and anal setae fine. Setal lengths: *1a* 80–100, *1b* 10–15, *1c* 10–12, *2b* 10–14, *2c* 18–20, *3a* 12–13, *3b* 12–17, *4a* 50–80, *4b* 12–21, *ag* 7–8, *g1* 8–9, *ps1* 12–14, *ps2* 25–27, *ps3* 9–10. Setae *g2* absent.

Legs (Figure 16). Setation (from coxae to tarsi): I 3–1–4–3–5–8(1), II 2–1–4–3–5–8(1), III 1–2–2–1–3–5, IV 1–0–1–0–3–5. Leg chaetotaxy similar to that of the female, except by trochanter IV nude; tarsi I–II each with one solenidion *ω”* (tarsi I 4–5 and tarsi II 5), and two eupathidia *pζ′*–*pζ”* (4–5, 5; 4–5, 4–5, respectively). Detail of the development of leg chaetotaxy in Table 1.

**Figure 17:**
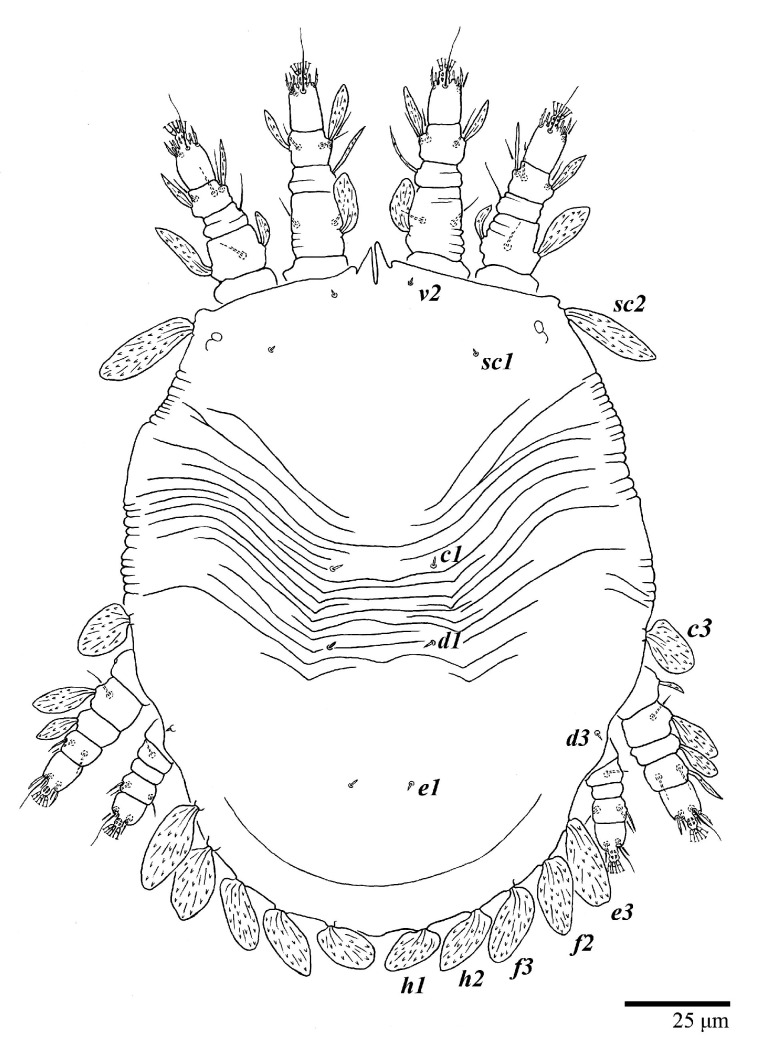
*Ultratenuipalpus parameekeri* Castro, Ochoa & Feres sp. nov. (Protonymph): dorsum, with detail of legs (unguinal setae *u′–u”* on tarsus I and II are not included in the drawing).

Protonymph (*n* = 3) (Figure 17)

Body measurements: distance between setae *v2*–*h1* 275–290, *sc2*–*sc2* 135–145; other measurements: *v2*–*v2* 32–35, *sc1*–*sc1* 80–83, *c1*–*c1* 40–43, *c3*–*c3* 185–195, *d1*–*d1* 35–38, *d3*–*d3* 150–155, *e1*–*e1* 22–25, *e3*–*e3* 120–130, *f2*–*f2* 110–115, *f3*–*f3* 90–95, *h1*–*h1* 30–33, *h2*–*h2* 62–65.

Dorsum (Figure 17). Anterior margin of prodorsum with a short median forked projection forming a short notch; pair of lateral body projections anterior and adjacent to setae *sc2* present. Prodorsal region smooth; region between setae *sc2*–*c3* with transverse striations and region posterior to setae *c3* smooth; dorsal setae similar to that of the female, except setae *c1*, *d1*, and *e1* short. Setal measurements: *v2* 2–3, *sc1* 2–3, *sc2* 40–44, *c1* 3–4, *c3* 24–25, *d1* 3–4, *d3* 3–4, *e1* 2–3, *e3* 30–32, *f2* 27–28, *f3* 24–26, *h1* 22–25, *h2* 24–25.

Gnathosoma. Palps similar to those of the female, setal formula: 0, 0, 2, 2; tibia with two setae, *d′* 4–5, *d″* 3–4, tarsus with one eupathidium 3–4 and one solenidion, 1 long. Ventral setae *m* 4–5; distance between setae *m–m* 10–12.

Venter. Cuticle covered with fine and mostly transverse striae. Coxal, genital and anal setae fine. Setal measurements: *1a* 65–67, *1b* 10–11, *1c* 9–12, *2c* 12–13, *3a* 10–13, *3b* 14–17, *ag* 6–7, *ps1* 7–9, *ps2* 13–15, *ps3* 7–8. Setae *2b*, *4a*, *4b*, *g1* and *g2* absent.

Legs (Figure 17). Setation (from coxae to tarsi): I 3–0–3–1–5–6(1), II 1–0–3–1–5–6(1), III 1–0–2–0–3–5, IV 0–0–1–0–3–3. Tarsi I–II each with one solenidion *ω”* 4–5 (for both tarsi I and tarsi II) and two eupathidia *pζ′–pζ”* (all 3–4). Detail of the development of leg chaetotaxy in Table 1.

**Figure 18:**
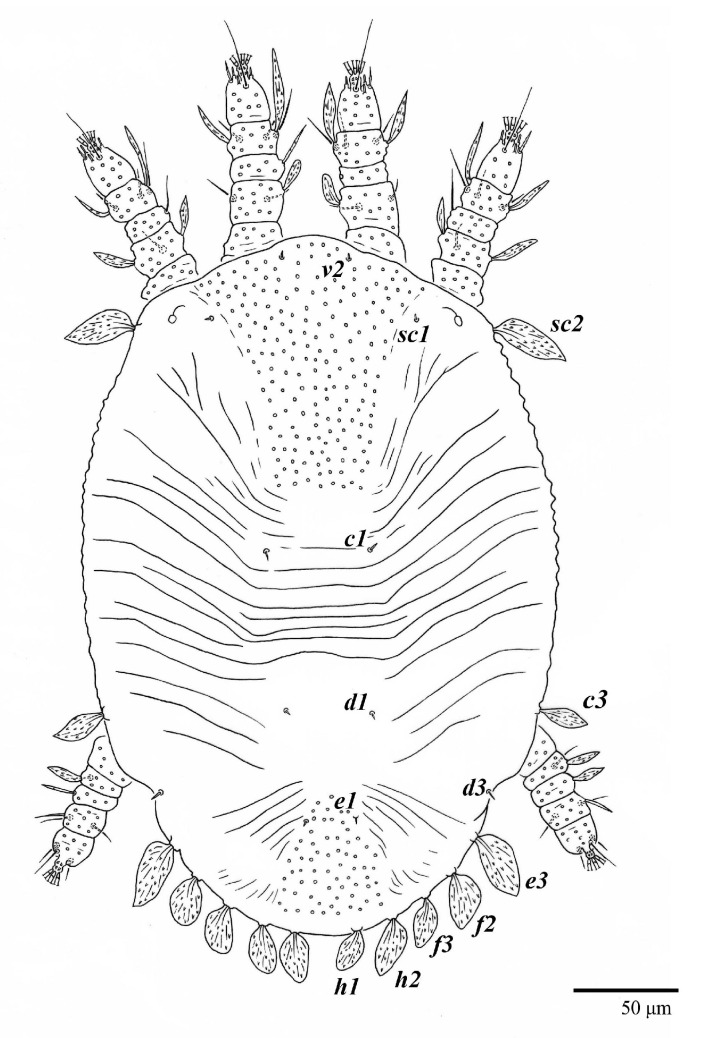
*Ultratenuipalpus parameekeri* Castro, Ochoa & Feres sp. nov. (Larva): dorsum, with detail of legs (unguinal setae *u′–u”* on tarsus I and II are not included in the drawing).

Larva (*n* = 3) (Figure 18)

Body measurements: distance between setae *v2*–*h1* 220–230, *sc2*–*sc2* 115–125; other measurements: *v2*–*v2* 22–25, *sc1*–*sc1* 70–73, *c1*–*c1* 32–38, *c3*–*c3* 140–150, *d1*–*d1* 30–38, *d3*–*d3* 110–115, *e1*–*e1* 16–18, *e3*–*e3* 100–105, *f2*–*f2* 86–88, *f3*–*f3* 67–70, *h1*–*h1* 20–23, *h2*–*h2* 40–45.

Dorsum (Figure 18). Prodorsal region with colliculated integument anteromedially; region between setae *sc2*–*c3* with oblique and transverse folds; pygidial region posterior to setae *e1* with colliculated integument; dorsal setae similar in general form to those of females except much smaller and setae *c1*, *d1*, and *e1* minute. Setal measurements: *v2* 2–3, *sc1* 1–2, *sc2* 25–26, *c1* 2–4, *c3* 16–18, *d1* 2–3, *d3* 2–3, *e1* 2–3, *e3* 22–23, *f2* 16–20, *f3* 15–18, *h1* 15–17, *h2* 16–17.

Gnathosoma. Palps similar to those of female, setal formula: 0, 0, 2, 2; tibia with two setae, *d′* 3–4, *d″* 5, tarsus with one eupathidium 3–4 and one minute solenidion, 1 long. Setae *m* absent.

Venter. Cuticle covered with fine and mostly transverse striae. Coxal, genital, and anal setae fine. Setal measurements: *1a* 55–65, *1b* 7–8, *3a* 10–11, *ps1* 5–7, *ps2* 10–11, *ps3* 5–6. Setae *1c*, *2b*, *2c*, *3b*, *4a*, *4b*, *ag*, *g1*, and *g2* absent.

Legs (Figure 18). Setation (from coxae to tarsi): I 2–0–3–1–5–6(1), II 0–0–3–1–5–6(1), III 0–0–2–1–3–3. Tarsi I–II each with one solenidion *ω”* 3–4 (for both tarsi I and II) and two eupathidia *pζ′–pζ”* (3–4, 3–4; 3–4, 3–4, respectively). Cuticle of all legs covered with colliculated sculpturing. Detail of the development of leg chaetotaxy in Table 1.

Etymology. The specific name *parameekeri* refers to the morphological similarity of this species and *U. meekeri* (De Leon), the type species of the genus.

Differential diagnosis. This new species resembles *Ultratenuipalpus meekeri* (De Leon) (herein redescribed) as they both have dorsal setae of a similar shape and length and the same leg and palp chaetotaxy in all developmental stages. These two species also share several other characteristics, such as the pair of lateral projections anterior to setae *sc2* and a single posterior projection between opisthosomal setae *h1*. However, the two species can be separated: the prodorsum is distinctly broader in adult females and males (measured at the widest point between setae *sc1* and *c1*) in *U. meekeri* (325–345) than *U. parameekeri* (290–315) (in females); notch in anterior forked projection is shorter in *U. meekeri* (8–13) than in *U. parameekeri* (20–27) (in females); *e3* is narrower and more lanceolate on male (and to a lesser extent on females) *U. meekeri* than in *U. parameekeri*; *l”* on ti I on *U. meekeri* is thicker than on *U. parameekeri*; *d* on fe II is longer and more falcate on female *U. meekeri* than on *U. parameekeri*; *c3* in larvae is narrower and more lanceolate in *U. meekeri* than *U. parameekeri*. In addition to the morphological differences, the molecular analyses confirmed that *U. parameekeri* and *U. meekeri* represent distinct species, with a 15.7% difference between their COI sequences.

DNA Barcoding. DNA was successfully amplified and the mitochondrial cytochrome C oxidase subunit I gene (COI) sequenced from one specimen of *U. parameekeri* collected on *Cyclosorus interruptus* (Thelypteridaceae) from Pindorama, São Paulo, Brazil; sequence data have been deposited in GenBank (https://www.ncbi.nlm.nih.gov/, accessed on 15 January 2023), with the following accession code: female, 398 base pairs (GenBank: OQ533138).

Type material examined. Holotype: female collected on ferns *Rumohra adiantiforme* (Dryopteridaceae) from Ilha do Cardoso, São Paulo, Brazil, 22 March 2017, coll. G.C.O. Piccoli (DZSJRP). Paratypes: 3 females, 1 protonymph, and 2 larvae, with the same data as the holotype (DZSJRP); 4 females, 3 males, 4 deutonymphs, 5 protonymphs, and 2 larvae collected on *Psychotria nuda* (Rubiaceae) from Ilha do Cardoso, São Paulo, Brazil, 22 March 2017, coll. G.C.O. Piccoli (DZSJRP); 2 females and 2 males collected on *P. nuda* from Ilha do Cardoso, São Paulo, Brazil, 22 March 2017, coll. G.C.O. Piccoli (NMNH); 4 females and 1 deutonymph collected on ferns *C. interruptus* from Pindorama, São Paulo, Brazil, 15 December 2002, coll. R. Kishimoto (DZSJRP).

Other material examined. 1 female and 1 larva collected on ferns *C. interruptus* from Pindorama, São Paulo, Brazil, 15 March 2003, coll. P. Demite (DZSJRP); 2 females, 2 deutonymphs, 3 protonymphs, and 1 larva collected on ferns *C. interruptus* from Pindorama, São Paulo, Brazil, 15 December 2002, coll. R. Kishimoto (DZSJRP); 2 females, 2 deutonymphs, 3 protonymphs, and 1 larva collected on ferns *C. interruptus* from Pindorama, São Paulo, Brazil, 15 March 2005, coll. P. Demite (DZSJRP, USNM).

### 3.2. Redescription of Ultratenuipalpus meekeri (De Leon, 1957)

*Tenuipalpus meekeri* De Leon: De Leon [13]—original designation
*Ultratenuipalpus meekeri* (De Leon): Mitrofanov [14]

Redescriptions [4,15,16,17,18,19].

Diagnosis. Female: As per genus, in addition to: prodorsal setae *v2*, *sc1* minute to short, and *sc2* large, flattened, obovate to ovate; dorsal opisthosoma with 10 pairs of setae (*f2* present); most of the dorsal opisthosomal setae large, flattened, obovate to ovate, except setae *d3* is distinctly short and *c3* is almost orbicular; pair lateral projections anterior to setae *sc2* and single posterior projection between opisthosomal setae *h1* present; palp four segmented, setal formula 0, 0, 2, 2. Male: Opisthosoma narrower than that of females, with a distinct transverse constriction (waist) between setae *d1* and *e1*; many dorsal setae similar to those of the female, except *c1* much smaller, *d1* and *e1* short to minute, and *v2* and *d3* longer. Tarsi I–II each with two solenidia (*ω′* paraxial and ventrolateral; *ω′* antiaxial); tarsus III with one solenidion *ω′* paraxial and ventrolateral. Immatures: with lateral body projections anterior to setae *sc2* present (except absent in larvae); single posterior projection between setae *h1* absent; dorsal setae similar in general form to those of the female, except *c1*, *d1*, and *e1* minute. Larvae with anterior margin colliculated and central prodorsum smooth; pygidial region of posterior opisthosoma with colliculated integument.

**Figure 19:**
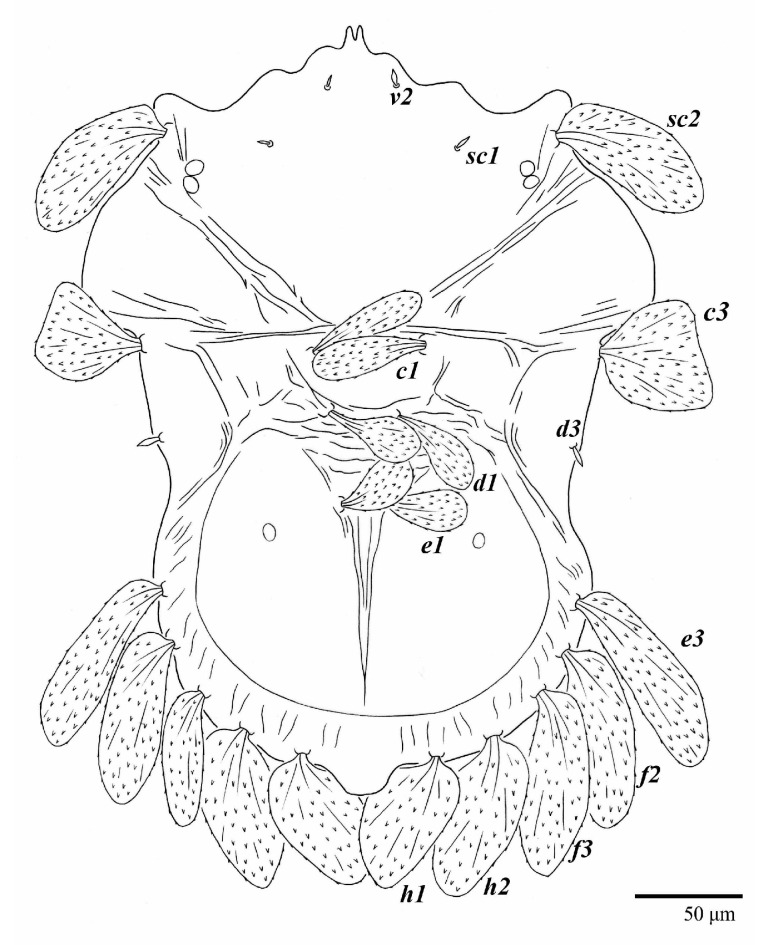
*Ultratenuipalpus meekeri* (De Leon). (Female, paratype): view of dorsum.

**Figure 20:**
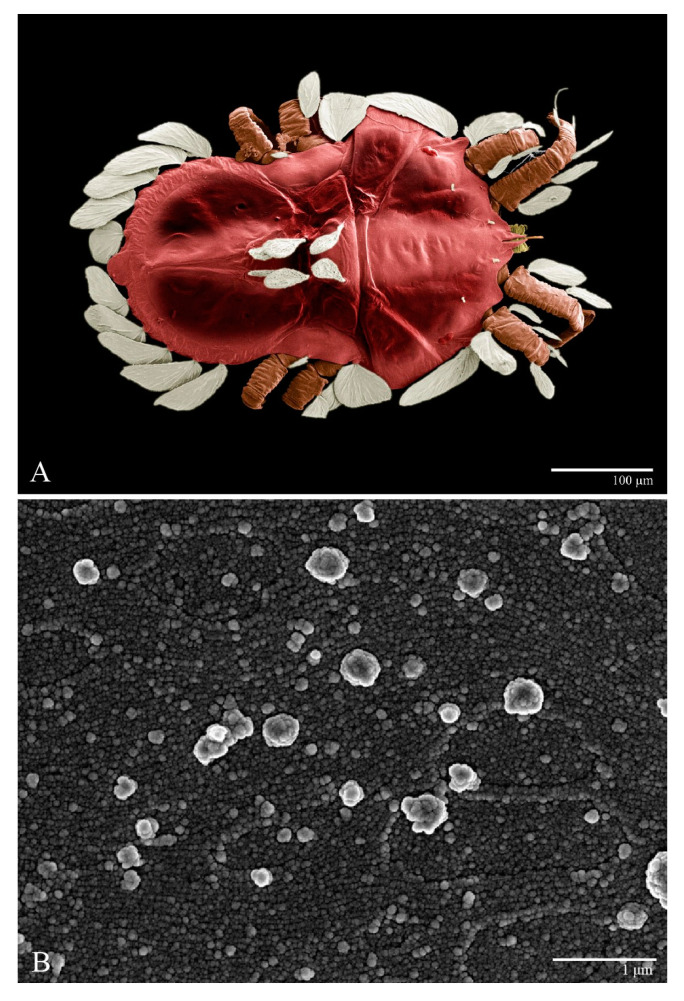
*Ultratenuipalpus meekeri* (De Leon). (Female): (**A**) dorsal view; (**B**) view of cuticular microplates on the dorsum.

**Figure 21:**
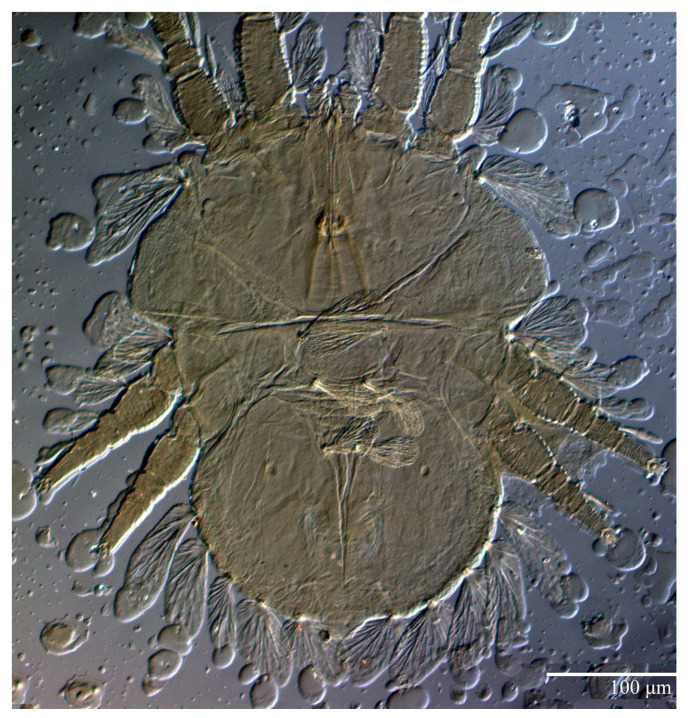
*Ultratenuipalpus meekeri* (De Leon). (Female, paratype): view of dorsum.

**Figure 22:**
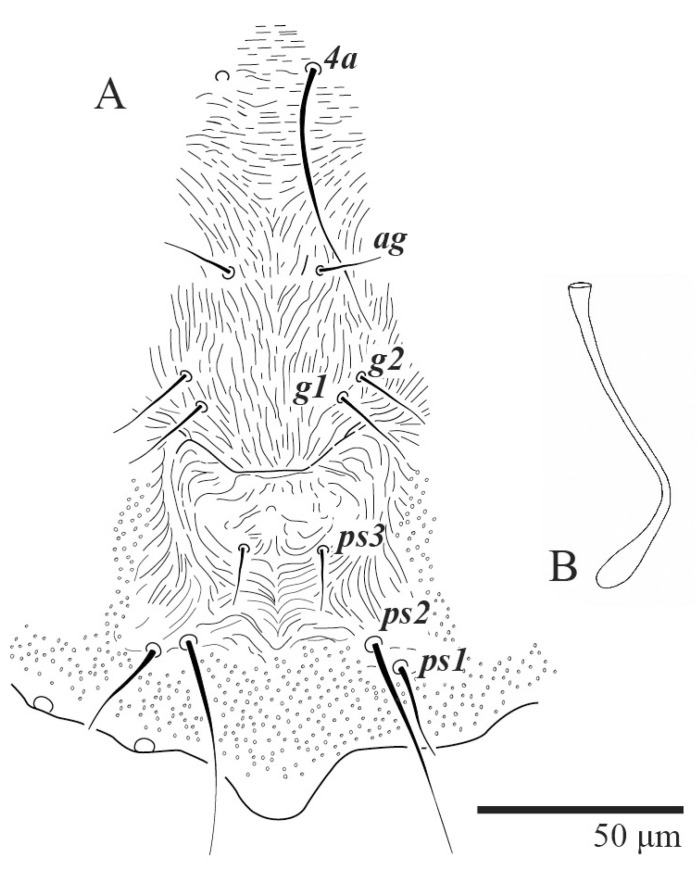
*Ultratenuipalpus meekeri* (De Leon). (Female, paratype): (**A**) posterior ventral opisthosoma; (**B**) spermatheca.

**Figure 23:**
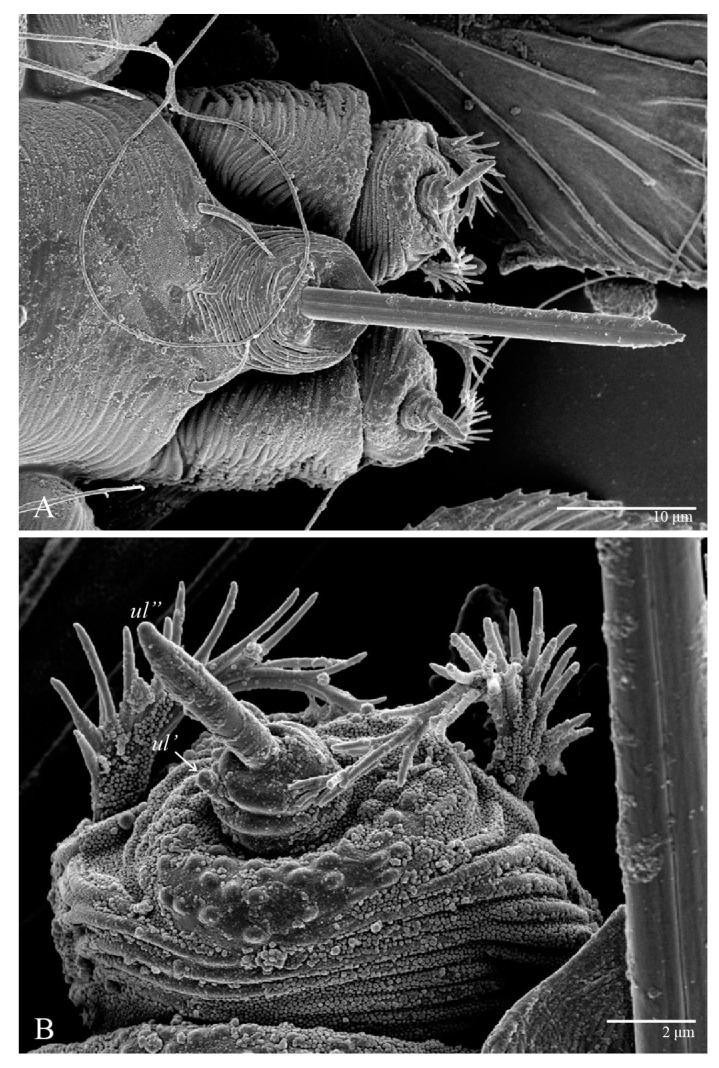
*Ultratenuipalpus meekeri* (De Leon). (Female): (**A**) view of ventral infracapitulum; (**B**) detail of palp; note the basal insertion of solenidion.

**Figure 24:**
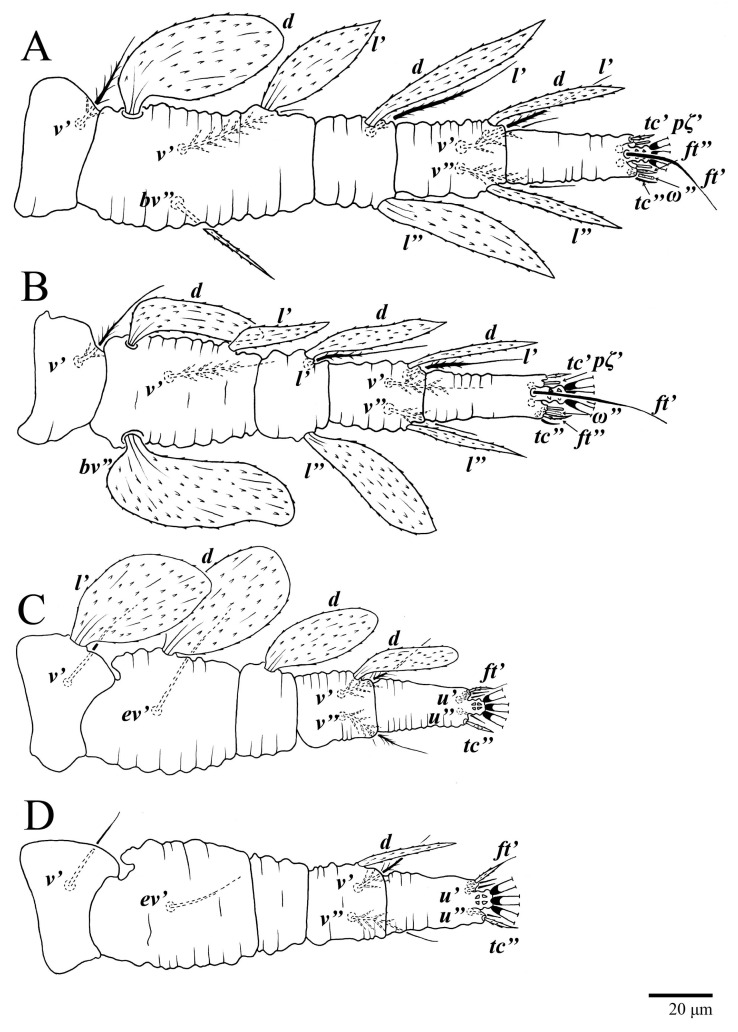
*Ultratenuipalpus meekeri* (De Leon). (Female, paratype): (**A**) leg I; (**B**) leg II; (**C**) leg III; (**D**) leg IV. (Right legs).

Female (*n* = 3) (Figure 19, Figure 20, Figure 21, Figure 22, Figure 23 and Figure 24)

Body measurements: distance between setae *v2*–*h1* 390 (375–390), *sc2*–*sc2* 230 (230–235); other measurements: *v2*–*v2* 42 (42–48), *sc1*–*sc1* 115 (115), *c1*–*c1* 65 (65–68), *c3*–*c3* 290 (260–290), *d1*–*d1* 43 (37–43), *d3*–*d3* 250 (235–250), *e1*–*e1* 28 (25–30), *e3*–*e3* 235 (230–240), *f2*–*f2* 225 (215–225), *f3*–*f3* 190 (185–195), *h1*–*h1* 72 (72–78), *h2*–*h2* 135 (130–140).

Dorsum (Figure 19, Figure 20 and Figure 21). Anterior margin of prodorsum with a short median forked projection forming a short notch 8 (8–13). Dorsum smooth, with pair of lateral projections anterior to setae *sc2* and a single projection between opisthosomal setae *h1* present. A pair of converging folds from the eyes to near the sejugal furrow on the prodorsum posterior margin. Prodorsal setae *v2* and *sc1* short to minute; *sc2* large, flattened elongated obovate (Figure 19 and Figure 20A); most opisthosomal setae similar to prodorsal setae *sc2*, except *d3* short. Setal measurements: *v2* 8 (4–8), *sc1* 4 (4–10), *sc2* 83 (83–94), *c1* 65 (65–69), *c3* 57 (54–57), *d1* 55 (55–58), *d3* 14 (14–15), *e1* 48 (39–48), *e3* 95 (92–95), *f2* 80 (80–84), *f3* 77 (77–83), *h1* 63 (62–66), *h2* 70 (70–73).

Venter (Figure 22A). Ventral integument weakly striate along central region and densely colliculated around lateral body margin; ventral, genital, and anal plates not developed, entire region membranous and distinctly plicate; ventral setae filiform, with coxal setae *1c*, *2c*, and *3b* barbed; setae *ps2* distinctly longer than *ps1*. Setal measurements: *1a* 105 (105–115), *1b* 19 (12–19), *1c* 29 (26–29), *2b* 27 (27–28), *2c* 47 (41–47), *3a* 20 (20–21), *3b* 43 (37–43), *4a* 105 (95–115), *4b* 26 (23–26), *ag* 15 (15–17), *g1* 19 (15–19), *g2* 17 (17–20), *ps1* 15 (12–15), *ps2* 48 (48–60), *ps3* 31 (23–31).

Gnathosoma (Figure 23). Palps four segmented, setal formula: 0, 0, 2, 2; tibia with two setae, *d′* 7 (7–11), *d″* 8 (7–8), tarsus with one eupathidium 5 (5) and one solenidion, 1 (1) long. Ventral setae *m* 7 (7–8); distance between setae *m*–*m* 15 (13–15).

Spermatheca (Figure 22B). Duct length ca. 70–85, terminating in smooth rounded bulb.

Legs (Figure 24). Setation (from coxae to tarsi): I 3–1–4–3–5–8(1), II 2–1–4–3–5–8(1), III 1–2–2–1–3–5, IV 1–1–1–0–3–5. Tarsi I–II each with one solenidion *ω”* 9 (8–9) (for both tarsi I and tarsi II) and two eupathidia *pζ′*–*pζ”* (7, 7; 7, 6–7, respectively); femur I with setae *d* obovate and *l′* broadly lanceolate; femur II with setae *d* elongate obovate to weakly falcate, *l′* lanceolate and *bv”* obovate to broadly falcate. Femora, genua, and tibiae with setae *d* inserted in lateral position. Detail of the development of leg chaetotaxy in Table 1.

Color (Figure 20A). The body is reddish with the central region becoming darker, eyes red, and legs orange. Dorsal body setae and legs setae are white.

**Figure 25:**
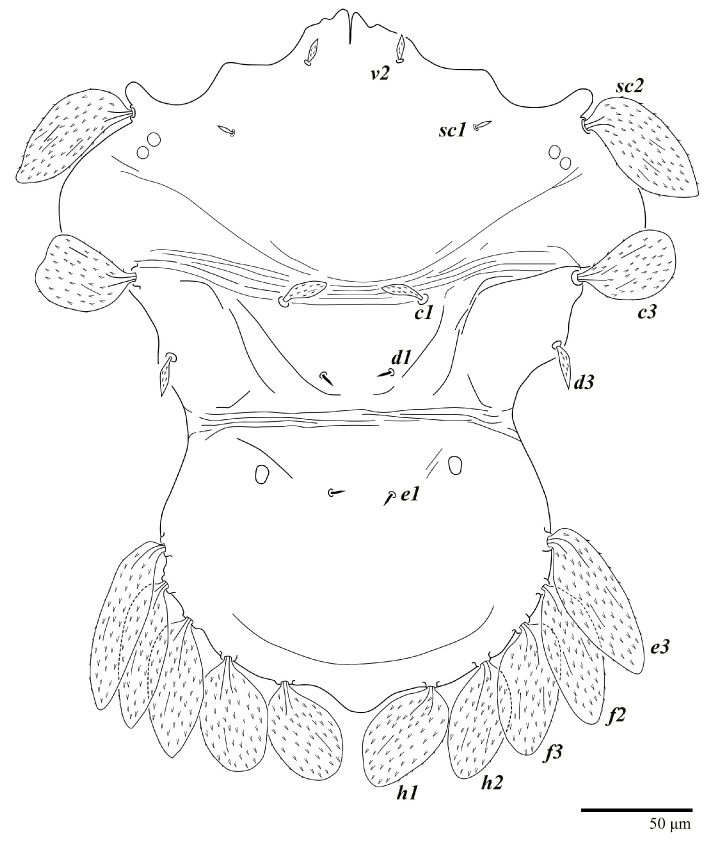
*Ultratenuipalpus meekeri* (De Leon). (Male, paratype): view of dorsum.

**Figure 26:**
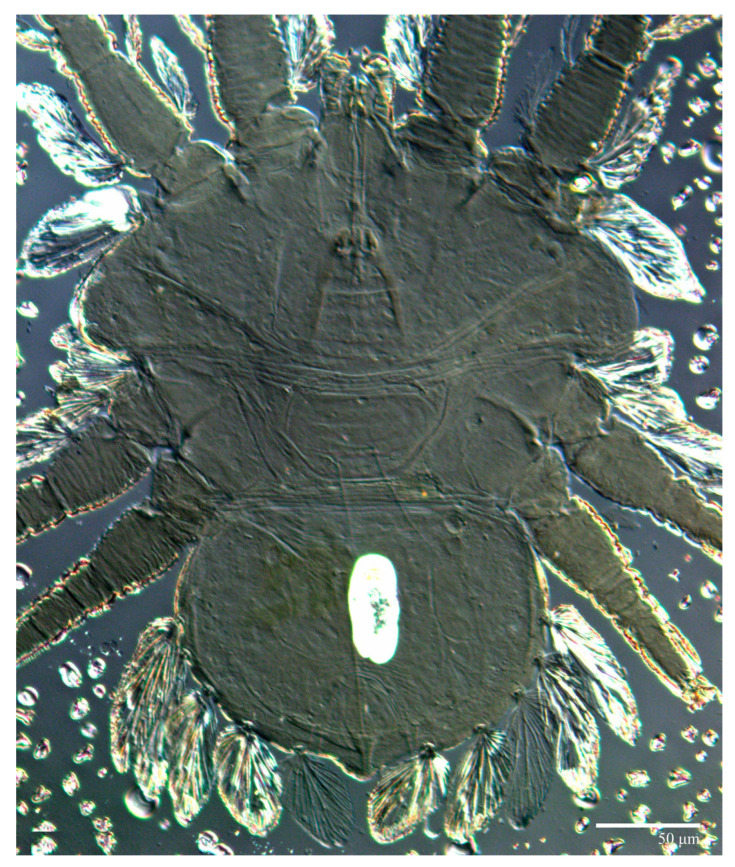
*Ultratenuipalpus meekeri* (De Leon). (Male, paratype): view of dorsum.

**Figure 27:**
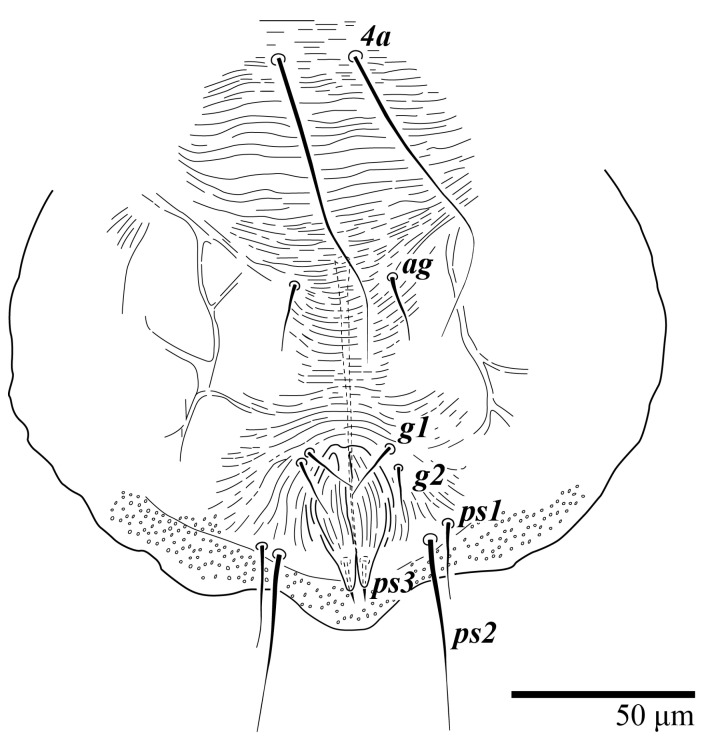
*Ultratenuipalpus meekeri* (De Leon). (Male, paratype): posterior ventral opisthosoma.

**Figure 28:**
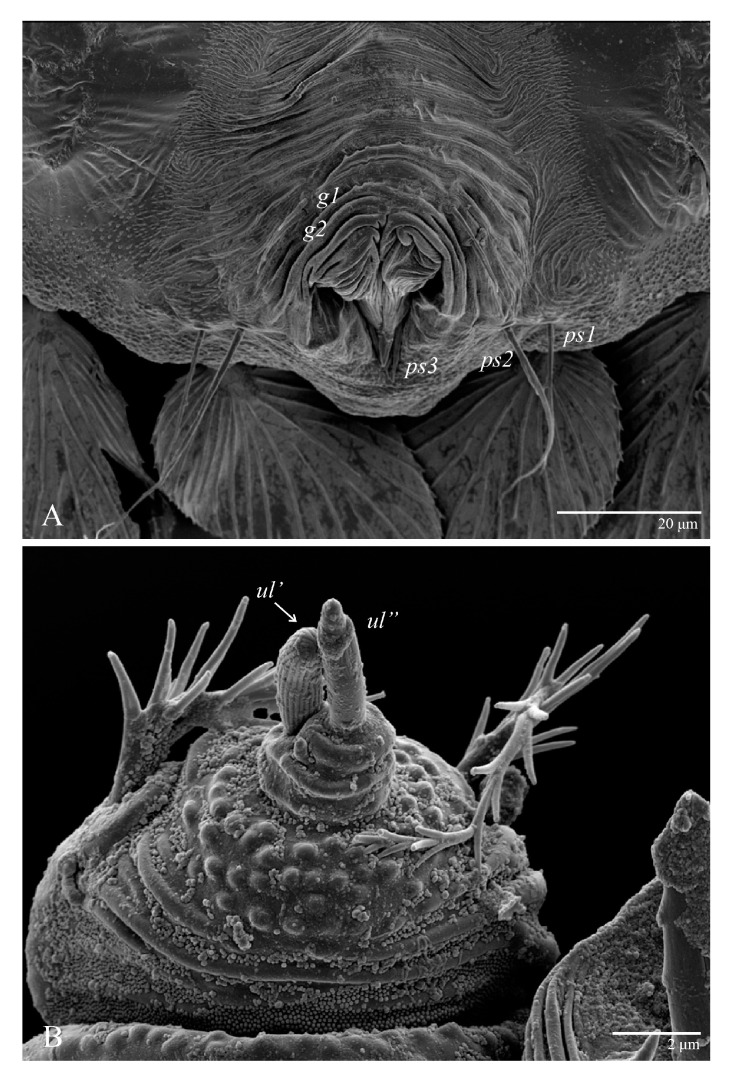
*Ultratenuipalpus meekeri* (De Leon). (Male): (**A**) posterior ventral opisthosoma; (**B**) detail of palp; note the basal insertion of solenidion.

**Figure 29:**
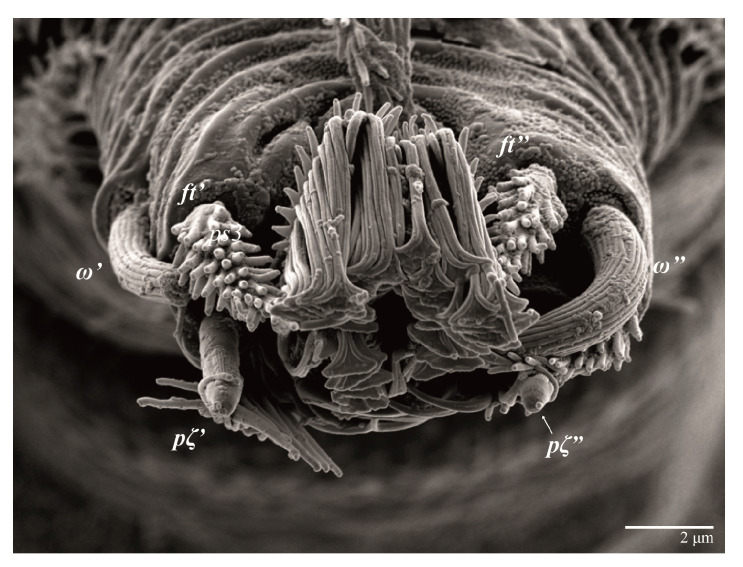
*Ultratenuipalpus meekeri* (De Leon). (Male): detail of tarsus II. Note the presence of solenidion *ω’* paraxial and ventrolateral.

Male (*n* = 1) (Figure 25, Figure 26, Figure 27, Figure 28 and Figure 29)

Body measurements: distance between setae *v2*–*h1* 280, *sc2*–*sc2* 210; other measurements: *v2*–*v2* 43, *sc1*–*sc1* 110, *c1*–*c1* 65, *c3*–*c3* 205, *d1*–*d1* 30, *d3*–*d3* 165, *e1*–*e1* 28, *e3*–*e3* 175, *f2*–*f2* 170, *f3*–*f3* 150, *h1*–*h1* 63, *h2*–*h2* 110.

Dorsum (Figure 25 and Figure 26). Anterior margin of prodorsum with a short median forked projection forming a short notch. Dorsum smooth, with pair lateral projections anterior to setae *sc2* and a single projection between opisthosomal setae *h1* present. Prodorsum with a pair of converging folds from the eyes to near the sejugal furrow on the posterior margin. Dorsal setae similar in general form to those of the female, except *c1*, *d1*, and *e1* small to minute, and *d3* longer. Setal measurements: *v2* 10, *sc1* 8, *sc2* 65, *c1* 23, *c3* 49, *d1* 6, *d3* 18, *e1* 5, *e3* 74, *f2* 70, *f3* 66, *h1* 53, *h2* 57.

Venter (Figure 27 and Figure 28A). Ventral integument weakly striated along central region and densely colliculated around lateral margin of body; ventral setae filiform; coxal setae *1c*, *2c*, and *3b* barbed; setae *ps2* distinctly longer than *ps1*; setae *ps3* thickened and inserted ventrodistally on elongated, tapered anal valves. Setal measurements: *1a* 85, *1b* 22, *1c* 28, *2b* 26, *2c* 35, *3a* 21, *3b* 35, *4a* 90, *4b* 23, *ag* 20, *g1* 19, *g2* 16, *ps1* 24, *ps2* 60, *ps3* 16.

Gnathosoma (Figure 28B). Palps four segmented, setal formula: 0, 0, 2, 2; tibia with two setae, *d′* 8, *d″* 7, tarsus with one eupathidium 5 and one solenidion 6. Ventral setae *m* 8; distance between setae *m*–*m* 14.

Legs. Setation (from coxae to tarsi): I 3–1–4–3–5–8(2), II 2–1–4–3–5–8(2), III 1–2–2–1–3–5(1), IV 1–1–1–0–3–5. Tarsi I–II (Figure 29) each with two solenidia (one abaxial, one adaxial), tarsi I *ω′* 12, *ω″* 9, tarsi II *ω′* 13, *ω″* 9, and two eupathidia *pζ′*–*pζ”* (all 6–7), and tarsus III with one solenidion (paraxial and ventrolateral) *ω′* 12. Leg setae similar to that of the female. Detail of the development of leg chaetotaxy in Table 1.

**Figure 30:**
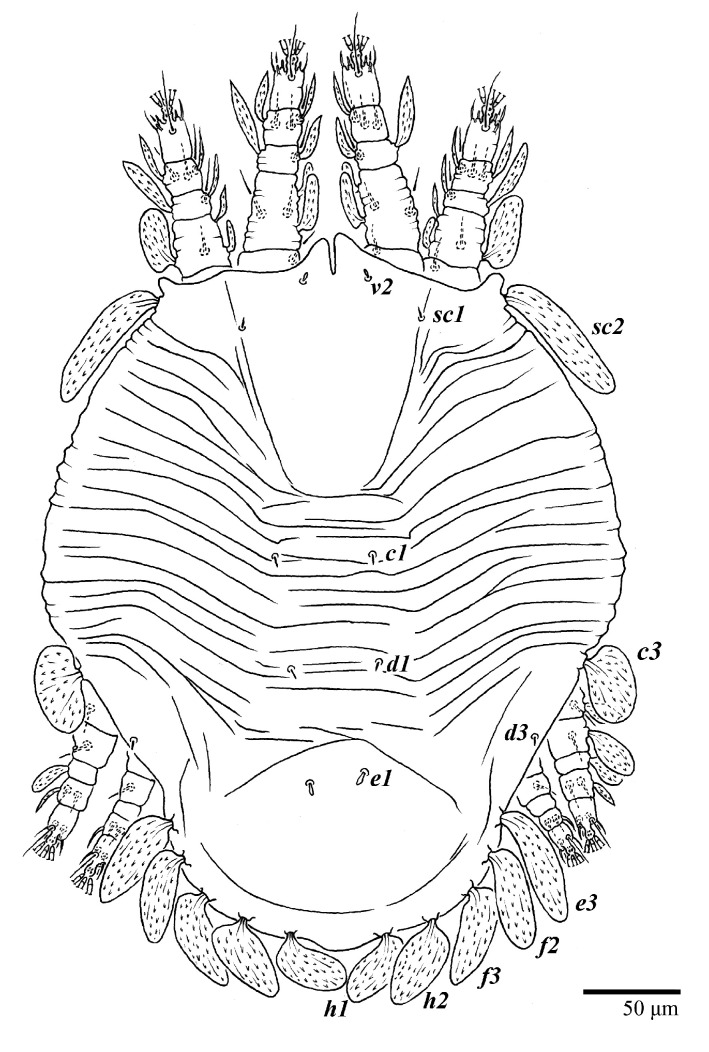
*Ultratenuipalpus meekeri* (De Leon). (Deutonymph, paratype): dorsum, with detail of legs (unguinal setae *u′–u”* on tarsus I and II are not included in the drawing).

**Figure 31:**
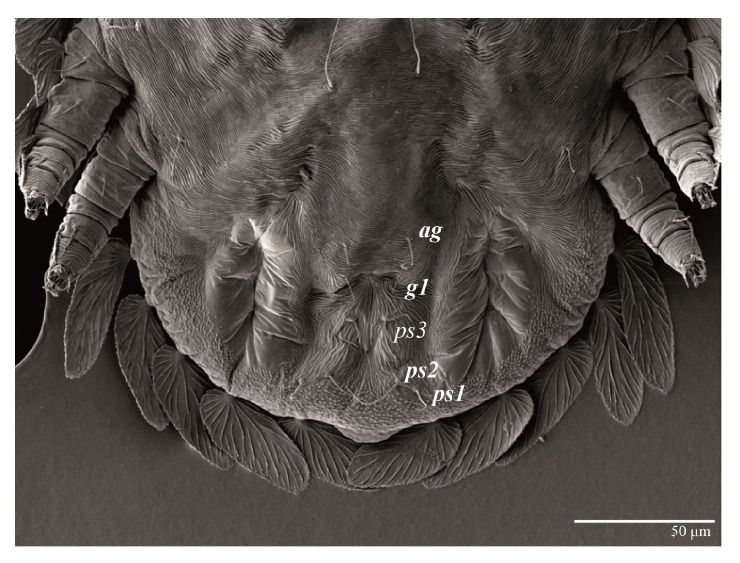
*Ultratenuipalpus meekeri* (De Leon). (Deutonymph): posterior ventral opisthosoma.

Deutonymph (*n* = 3) (Figure 30 and Figure 31)

Body size measurements: distance between setae *v2*–*h1* 310–350, *sc2*–*sc2* 170–190; other measurements: *v2*–*v2* 37–40, *sc1*–*sc1* 95–105, *c1*–*c1* 43–53, *c3*–*c3* 220–270, *d1*–*d1* 45–55, *d3*–*d3* 200–225, *e1*–*e1* 25–30, *e3*–*e3* 160–180, *f2*–*f2* 145–170, *f3*–*f3* 120–145, *h1*–*h1* 40–55, *h2–h2* 83–105.

Dorsum (Figure 30). Anterior margin of prodorsum with a short median forked projection forming a short notch; a pair of body projections anterior and adjacent to setae *sc2* present; posterior projection between setae *h1* absent. Prodorsum with central region smooth; region between setae *sc2*–*c3* with transverse folds and plicae; region posterior to setae *e1* smooth. Dorsal setae similar in general form to those of the female, except setae *c1*, *d1*, and *e1* are short to minute. Setal measurements: *v2* 3–5, *sc1* 4–5, *sc2* 64–78, *c1* 6–12, *c3* 36–41, *d1* 5–7, *d3* 5–7, *e1* 4–8, *e3* 52–64, *f2* 51–55, *f3* 47–55, *h1* 36–42, *h2* 43–51.

Gnathosoma. Palps similar to those of female, setal formula: 0, 0, 2, 2; tibia with two setae, *d′* 6–7, *d″* 5–6, tarsus with one eupathidium 4–5 and one minute solenidion 1. Ventral setae *m* 5–7; distance between setae *m*–*m* 12–13.

Venter (Figure 31). Cuticle covered with fine and mostly transverse striae; with band of a colliculated cuticle around posterior body margin. Coxal, genital, and anal setae fine. Setal lengths: *1a* 75–90, *1b* 9–12, *1c* 11–15, *2b* 10–18, *2c* 13–16, *3a* 10–15, *3b* 16–17, *4a* 60–80, *4b* 11–12, *ag* 11–15, *g1* 8–11, *ps1* 9–10, *ps2* 27–33, *ps3* 15–17. Setae *g2* absent.

Legs (Figure 30). Setation (from coxae to tarsi): I 3–1–4–3–5–8(1), II 2–1–4–3–5–8(1), III 1–2–2–1–3–5, IV 1–0–1–0–3–5. Leg chaetotaxy similar to that of the female, except by trochanter IV nude; tarsi I–II each with one solenidion *ω”* (tarsi I 5–6 and tarsi II 5, 6) and two eupathidia *pζ′*–*pζ”* (5–6, 5–6; 5, 5 respectively). Detail of the development of leg chaetotaxy in Table 1.

**Figure 32:**
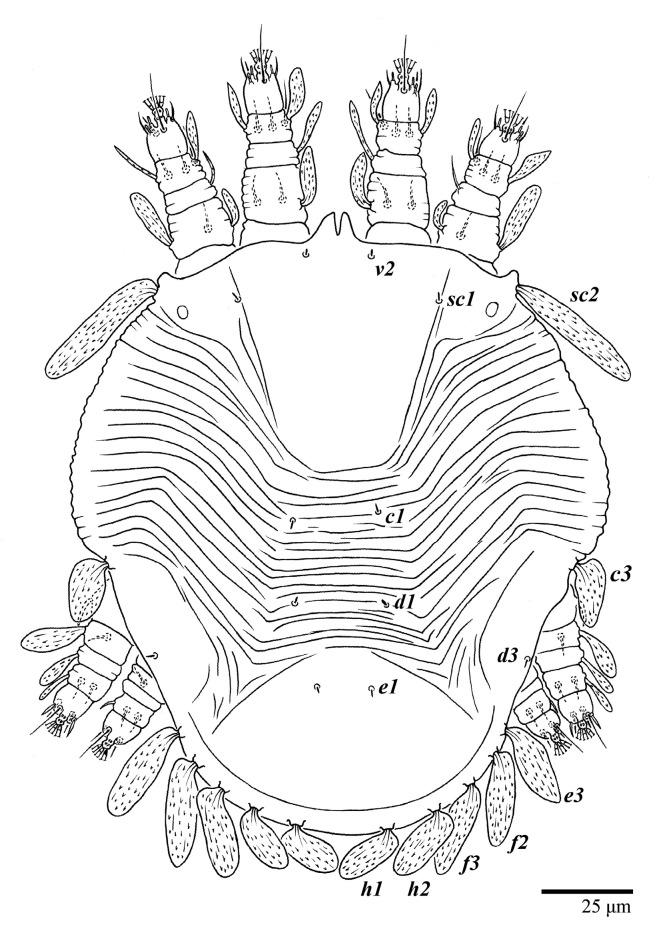
*Ultratenuipalpus meekeri* (De Leon). (Protonymph, paratype): dorsum, with detail of legs (unguinal setae *u′–u”* on tarsus I and II are not included in the drawing).

Protonymph (*n* = 1) (Figure 32)

Body size measurements: distance between setae *v2*–*h1* 230, *sc2*–*sc2* 150; other measurements: *v2*–*v2* 28, *sc1*–*sc1* 85, *c1*–*c1* 35, *c3*–*c3* 190, *d1*–*d1* 25, *d3*–*d3* 155, *e1*–*e1* 23, *e3*–*e3* 130, *f2*–*f2* 120, *f3*–*f3* 100, *h1*–*h1* 38, *h2*–*h2* 73.

Dorsum (Figure 32). Anterior margin of prodorsum with a short median forked projection forming a short notch; a pair of lateral body projections anterior and adjacent to setae *sc2* present; posterior projection between setae *h1* absent. Prodorsum with central region smooth; region between setae *sc2*–*c3* with transverse folds and plicae; region posterior to setae *e1* smooth; dorsal setae similar in general form to those of the female, except setae *c1*, *d1*, and *e1* short to minute. Setal measurements: *v2* 3, *sc1* 2, *sc2* 54, *c1* 4, *c3* 27, *d1* 4, *d3* 4, *e1* 4, *e3* 45, *f2* 38, *f3* 35, *h1* 30, *h2* 35.

Gnathosoma. Palps similar to those of the female, setal formula: 0, 0, 2, 2; tibia with two setae, *d′* 4, *d″* 4, tarsus with one eupathidium 3 and one minute solenidion, 1 long. Ventral setae *m* 5; distance between setae *m*–*m* 12.

Venter. Cuticle covered with fine and mostly transverse striae. Coxal, genital, and anal setae fine. Setal measurements: *1a* 70, *1b* 9, *1c* 8, *2c* 13, *3a* 14, *3b* 11, *ag* 10, *ps1* 8, *ps2* 16, *ps3* 10. Setae *2b*, *4a*, *4b*, *g1*, and *g2* absent.

Legs (Figure 32). Setation (from coxae to tarsi): I 3–0–3–1–5–6(1), II 1–0–3–1–5–6(1), III 1–0–2–1–3–5, IV 0–0–1–0–3–3. Tarsi I–II each with one solenidion *ω”* 4 (for both tarsi I and tarsi II) and two eupathidia *pζ′*–*pζ”* (all 5). Detail of the development of leg chaetotaxy in Table 1.

**Figure 33:**
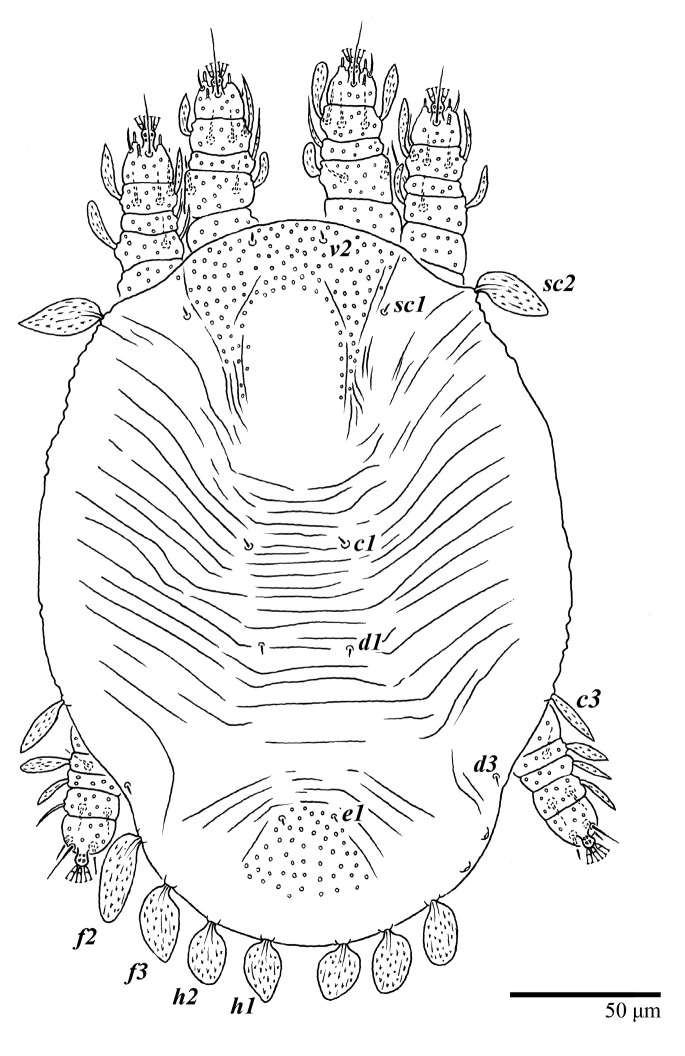
*Ultratenuipalpus meekeri* (De Leon). (Larva, paratype): dorsum, with detail of legs (unguinal setae *u′–u”* on tarsus I and II are not included in the drawing).

Larva (*n* = 1) (Figure 33)

Body size measurements: distance between setae *v2*–*h1* 225, *sc2*–*sc2* 120; other measurements: *v2*–*v2* 33, *sc1*–*sc1* 68, *c1*–*c1* 33, *c3*–*c3* 160, *d1*–*d1* 30, *d3*–*d3* 120, *e1*–*e1* 18, *e3*–*e3* 115, *f2*–*f2* 105, *f3*–*f3* 88, *h1*–*h1* 23, *h2*–*h2* 58.

Dorsum (Figure 33). Prodorsal region with broad band of a colliculated integument anteromedially between setae *sc1*; region between setae *sc2*–*c3* with oblique and transverse folds and plicae; pygidial region posterior to setae *e1* with a small region of colliculated integuments; dorsal setae similar in general form to those of the female, except setae *c1*, *d1*, and *e1* are short to minute. Setal measurements: *v2* 3, *sc1* 3, *sc2* 28, *c1* 4, *c3* 23, *d1* 2, *d3* 3, *e1* 3, *e3* missing, *f2* 30, *f3* 25, *h1* 21, *h2* 21.

Gnathosoma. Palps similar to those of female, setal formula: 0, 0, 2, 2; tibia with two setae, *d′* 3, *d″* 5, tarsus with one eupathidium 3 and one minute solenidion, 1 long. Setae *m* absent.

Venter. Cuticle covered with fine and mostly transverse striae. Coxal, genital, and anal setae fine. Setal measurements: *1a* 50, *1b* 7, *3a* 10, *ps1* 7, *ps2* 11, *ps3* 6. Setae *1c*, *2b*, *2c*, *3b*, *4a*, *4b*, *ag*, *g1*, and *g2* absent.

Legs (Figure 33). Setation (from coxae to tarsi): I 2–0–3–1–5–6(1), II 0–0–3–1–5–6(1), III 0–0–2–1–3–3. Tarsi I–II each with one solenidion *ω”* 3 (for both tarsi I and II) and two eupathidia *pζ′*–*pζ”* (5, 5; 4, 4, respectively). Cuticles of all legs covered with colliculated cuticles. Detail of the development of leg chaetotaxy in Table 1.

Remarks. The new specimens examined in this study have similar body and setal measurements to those of the type specimens. In addition, the palp and leg chaetotaxy of those specimens match those of the type specimens.

DNA Barcoding. DNA was successfully amplified and the mitochondrial cytochrome C oxidase subunit I gene (COI) sequenced from one specimen of *U. meekeri* collected on *Acrostichum danaeifolium* (Pteridaceae) from Tecpan de Galeana, Guerrero State, Mexico; sequence data have been deposited in GenBank (https://www.ncbi.nlm.nih.gov/, accessed on 15 January 2023), with the following accession code: female, 446 base pairs (GenBank: OQ533137).

Type material examined: Holotype: the female collected on a fern in a mangrove swamp, from San Blas, Nayarit State, Mexico, 21 March 1957, coll. D. De Leon, was deposited in the Museum of Comparative Zoology (MCZ), Harvard University. Paratypes: 2 females, 1 male, 3 deutonymphs, 1 protonymph, and 1 larva, with the same data as the holotype, were deposited in the National Insect and Mite Collection, National Museum of Natural History (NMNH), Smithsonian Institution.

Other material examined: Non-type material: 5 females collected on ferns *A. danaeifolium* in a mangrove swamp, from Tecpan de Galeana, Guerrero State, **Mexico**, 5 September 2017, coll. G. Otero-Colina (USNM, DZJSRP).

## 4. Discussion

### 4.1. Ontogeny

Studies on possible patterns of ontogenetic development of chaetotaxy provide information potentially useful for understanding mite taxonomy, phylogeny, and biology [20]. The family Tenuipalpidae has the highest number of ontogenetic studies among all Trombidiformes mites [21], with ontogenetic data available for 60 species in 20 genera [22]. However, ontogenetic development is known only for one species of *Ultratenuipalpus*, *U. jubatus* Otley, Beard & Seeman [2,22]. Here, we discuss the ontogeny of the two species, *U. parameekeri* and *U. meekeri*, which share the same pattern of additions of leg setae (Table 1).

Trochanters. Setae *v′* are added to trochanters I, II, and III in the deutonymph and on trochanter IV in the adults. This is the standard pattern for other flat mites [22,23,24], and also for Tetranychidae [10]. Setae *l′* are added to trochanters III in the protonymph, and this addition also occurs in *U. jubatus*; although the expression of setae *l′* and *v′* varies within the family, this pattern has been commonly reported [2,22,23,25,26].

Femora. Setae *l′* on femora I and II are added in the deutonymph. The expression of setae *l′* on the deutonymph also occurs in *U. jubatus* and in many species of *Tenuipalpus* [2,22,23]. Setae *d* and *ev′* are present on femora III in the larva. This pattern is common in the Tenuipalpidae [22], but in *U. jubatus*, the addition of setae *ev′* is delayed until the protonymph. Setae *ev′* are added on the femora IV in the protonymph of *U. parameekeri* and *U. meekeri* (n.b., femora IV are not nude as described for *U. meekeri* in [17] and in the keys of [1,2]).

Genua. There is great variation in the chaetotaxy of genua I and II in the Tenuipalpidae [22,23]. Here, setae *l′* is present on genua I and II in the larva, and setae *d* and *l”* are added on genua I and II in the deutonymph. This pattern also occurs in *U. jubatus* and is common in the *Tenuipalpus* [22,23]. Setae *l′* is present on the genu III in the larva of the new species, and the same pattern occurs in *U. jubatus*; while many species of *Tenuipalpus* add setae *l′* or *d* on genu II in the deutonymph [22,23].

Tibiae. Although the number of tibial setae varies across the family, there are no post-larval additions made to the tibiae in the Tenuipalpidae [10]. Here, the number of tibial setae for both species is 5–5–3–3, as is also seen on *U. jubatus*, whereas setae *l′* are suppressed on tibiae III and IV on *U. avarua* Xu, Fan & Zhang [1,2,22].

Tarsi. *Ultratenuipalpus parameekeri* and *U. meekeri* have a pair of tectal setae added to tarsi I–III in the protonymph, as occurs in *U. jubatus*. However, many species of the *Tenuipalpus* added these setae in the deutonymph. As is the case for many additions to leg IV, the addition of the tectal setae on tarsi IV is delayed to the deutonymph. This same pattern also occurs in *U. jubatus*, while many *Tenuipalpus* add tectal setae to tarsi IV in the adults [22,23].

The solenidion *ω′* is added on each tarsi I–III in males of *U. parameekeri* and *U. meekeri*. Similarly, the male of *U. jubatus* has this solenidion added to tarsus I and II, but not to tarsus III [2]. This characteristic also occurs in other tenuipalpid genera, such as *Tenuipalpus*, *Prolixus*, and *Acaricis* [26,27,28], with some species of *Acaricis* also bearing one solenidion *ω’* on tarsus IV.

In response to the detailed work of Lindquist [10] on the patterns of setal additions to the legs in the family Tetranychidae, ontogenetic studies regarding the family Tenuipalpidae have received increasing attention in recent years. For example, the genus *Raoiella* has ontogenetic data available for 13 of the 22 known species [22]. However, despite this increase in attention, there are still many tenuipalpid genera that have received little or no such focused research [22], such as the *Ultratenuipalpus*. Only three of the 26 known species of the *Ultratenuipalpus* have so far been studied ontogenetically, and it is one of the genera that should receive priority in future studies. Filling these gaps may allow an adequate comparison of ontogenetic data between species and genera of the flat mite family, and as Lindquist [10] suggests, may contribute to our further understanding of the superfamily as a whole.

### 4.2. Distribution, Taxonomy and Systematic

The genus *Ultratenuipalpu*s is known from all zoogeographic regions of the world with the exception of the Nearctic and Western Palearctic regions [2,3,4]. To date, six species of the *Ultratenuipalpu*s have been described from the Neotropical region: the new species herein described from Brazil (which represents the first record of the genus for the country), in addition to two species from Mexico (*U. meekeri* and *U. younguisti* Baker & Tuttle) and three species from Chile (*U. acharis* (Gonzalez), *U. canelae* (Gonzalez), and *U. charlini* (Gonzalez)) [3,29].

According to Beard et al. [2], the presence or absence of opisthosomal setae *f2* may indicate a biogeographic pattern within the genus. Those species that lack *f2* show a putative Gondwanan distribution, being found in Chile, Australia, New Zealand, and the Cook Islands. Those species with setae *f2* present are found in China, the Philippines, Mexico, and now with the new species herein described, in Brazil. The unique exception for this pattern is *U. younguisti,* which lacks the setae *f2* and was described from Mexico (based on specimens intercepted in the USA).

The presence of a pair of lateral body projections anterior to setae *sc2* in some species of the *Ultratenuipalpus* (e.g., *U. meekeri*, *U. parameekeri*, and *U. avarua*) and the *Tenuipalpus sensu stricto* group could indicate a strong relationship between these two genera. Within the *Ultratenuipalpus*, the presence of a single posterior body projection between the setae *h1* may be an important character for separating a subgrouping, since it is shared by at least six species of the genus: *U. avarua*, *U. hainanensis* (Wang), *U. lacorpuzrarosae* Rimando, *U. meekeri*, *U. parameekeri*, and *U. umtataensis* Meyer.

## 5. Conclusions

The study of body morphology, spermathecae, geographic distribution, and plant associations will allow a broader and deeper understanding of the internal relationships within the genus *Ultratenuipalpus*, as well its relationships with other related genera (e.g., *Tenuipalpus*, *Extenuipalpus*, *Acaricis*, and *Prolixus*). In addition, we believe that the study of possible patterns of ontogenetic additions of leg setae can provide important insights into the systematics and origin of these taxa.

## Figures and Tables

**Table 1:** Additions of leg setae during ontogeny in both *Ultratenuipalpus parameekeri* Castro, Ochoa & Feres and *Ultratenuipalpus meekeri* (De Leon). The stage in which each seta first appears is indicated. Setae in parentheses represent pairs.

	Coxa	Trochanter	Femur	Genu	Tibia	Tarsi
Leg I						
Larva	*1b*	-	*d*, *v′*, *bv”*	*l′*	*d*, (*v*), (*l*)	(*u*), (*pζ*), (*ft*), *ω”*
Protonymph	*1c*	-	-	-	-	-
Deutonymph	-	*v′*	*l′*	*d, l”*	-	(*tc*)
Female/male	-	-	-	-	-	*ω’* ^1^
Leg II						
Larva	-	-	*d*, *v′*, *bv”*	*l′*	*d*, (*v*), (*l*)	(*u*), (*pζ*), (*ft*), *ω”*
Protonymph	*2c*	-	-	-	-	-
Deutonymph	*2b*	*v′*	*l’*	*d, l”*	-	(*tc*)
Female/male	-	-	-	-	-	*ω’* ^1^
Leg III						
Larva	-	-	*d*, *ev′*	*l’*	*d*, (*v*)	(*u*), *ft′*
Protonymph	*3b*	*l′*	-	-	-	-
Deutonymph	-	*v′*	-	-	-	-
Female/male	-	-	-	-	-	(*tc*), *ω′* ^1^
Leg IV						
Protonymph	-	-	*ev′*	-	*d*, (*v*)	(*u*), *ft′*
Deutonymph	*4b*	-	-	-	-	-
Female/male	-	*v′*	-	-	-	(*tc*)

^1^ Solenidion *ω’* added only on tarsi I, II, and III in the males.

## Data Availability

All data are available in this paper.
